# Reciprocal Regulation of Protein Synthesis and Carbon Metabolism for Thylakoid Membrane Biogenesis

**DOI:** 10.1371/journal.pbio.1001482

**Published:** 2013-02-12

**Authors:** Alexandra-Viola Bohne, Christian Schwarz, Marco Schottkowski, Michael Lidschreiber, Markus Piotrowski, William Zerges, Jörg Nickelsen

**Affiliations:** 1 Molecular Plant Sciences, Ludwig-Maximilians-University Munich, Planegg-Martinsried, Germany; 2 Biology Department and Centre for Structural and Functional Genomics, Concordia University, Montreal, Quebec, Canada; 3 Department of Plant Physiology, Ruhr-University Bochum, Bochum, Germany; Kyoto University, Japan

## Abstract

Metabolic control of gene expression coordinates the levels of specific gene products to meet cellular demand for their activities. This control can be exerted by metabolites acting as regulatory signals and/or a class of metabolic enzymes with dual functions as regulators of gene expression. However, little is known about how metabolic signals affect the balance between enzymatic and regulatory roles of these dual functional proteins. We previously described the RNA binding activity of a 63 kDa chloroplast protein from *Chlamydomonas reinhardtii*, which has been implicated in expression of the *psbA* mRNA, encoding the D1 protein of photosystem II. Here, we identify this factor as dihydrolipoamide acetyltransferase (DLA2), a subunit of the chloroplast pyruvate dehydrogenase complex (cpPDC), which is known to provide acetyl-CoA for fatty acid synthesis. Analyses of RNAi lines revealed that DLA2 is involved in the synthesis of both D1 and acetyl-CoA. Gel filtration analyses demonstrated an RNP complex containing DLA2 and the chloroplast *psbA* mRNA specifically in cells metabolizing acetate. An intrinsic RNA binding activity of DLA2 was confirmed by in vitro RNA binding assays. Results of fluorescence microscopy and subcellular fractionation experiments support a role of DLA2 in acetate-dependent localization of the *psbA* mRNA to a translation zone within the chloroplast. Reciprocally, the activity of the cpPDC was specifically affected by binding of *psbA* mRNA. Beyond that, in silico analysis and in vitro RNA binding studies using recombinant proteins support the possibility that RNA binding is an ancient feature of dihydrolipoamide acetyltransferases. Our results suggest a regulatory function of DLA2 in response to growth on reduced carbon energy sources. This raises the intriguing possibility that this regulation functions to coordinate the synthesis of lipids and proteins for the biogenesis of photosynthetic membranes.

## Abstract 2

A subunit of the chloroplast pyruvate dehydrogenase complex, which serves as a metabolic enzyme, also has a dual function as an RNA-binding protein and influences mRNA translation.

## Author Summary

Metabolic control of gene expression coordinates the levels of specific gene products to meet cellular demand for their activities. This control can be exerted by metabolites acting as regulatory signals on a class of metabolic enzymes with dual functions as regulators of gene expression. However, little is known about how metabolic signals affect the balance between enzymatic and regulatory roles of these proteins. Here, we report an example of a protein with dual functions in gene expression and carbon metabolism. The chloroplast pyruvate dehydrogenase complex is well-known to produce activated di-carbon precursors for fatty acid, which is required for lipid synthesis. Our results show that a subunit of this enzyme forms ribonucleoprotein particles and influences chloroplast mRNA translation. Conversely, RNA binding affects pyruvate dehydrogenase (metabolic) activity. These findings offer insight into how intracellular metabolic signaling and gene expression are reciprocally regulated during membrane biogenesis. In addition, our results suggest that these dual roles of the protein might exist in evolutionary distant organisms ranging from cyanobacteria to humans.

## Introduction

Accumulating evidence suggests that metabolism and gene expression are tightly linked. For instance, changes in metabolite levels affect protein modification, for example by acetylation or N-glycosylation, which in turn influences signal transduction and gene expression [1]–[3]. In line with this, several metabolic enzymes functioning in diverse pathways were found to possess unexpected RNA-binding properties by which they are proposed to regulate gene expression and other cellular processes (reviewed in [4],[5]). Often these proteins represent key enzymes of metabolic pathways, which make them particularly suitable to coordinate distinct biochemical pathways in response to changes in metabolism.

In eukaryotic organisms, photosynthesis is performed in endosymbiotically acquired organelles, the chloroplasts. Within chloroplasts, the light-driven reactions of photosynthesis take place in thylakoid membranes, which represent a highly organized system of lipid membranes and embedded multisubunit protein complexes. These complexes include photosystem I (PSI) and photosystem II (PSII), the cytochrome *b_6_f* complex, and the chloroplastic ATP synthase.

The biogenesis of thylakoid membranes requires the synthesis of both lipids and proteins. Major lipids include two glycolipids, monogalactosyl diacylglycerol (MGDG) and digalactosyl diacylglycerol (DGDG), the synthesis of which necessitates acetyl-CoA for fatty acid production within the chloroplast (reviewed in [6],[7]). This acetyl-CoA is mainly generated from pyruvate by the chloroplast pyruvate dehydrogenase complex (cpPDC), which—like its mitochondrial counterpart (mtPDC)—is a megadalton complex consisting of multiple copies of three subunits; a pyruvate dehydrogenase (E1), a dihydrolipoamide acetyltransferase (DLA, E2), and a dihydrolipoyl dehydrogenase (E3) [8]–[11]. The decarboxylation of pyruvate by cpPDC is a central reaction in chloroplast carbon metabolism and is regulated by light, Mg^2+^, and feedback inhibition by acetyl-CoA and NADH (for reviews, see [9],[11]).

The biogenesis of thylakoid membranes also necessitates the synthesis of polypeptides and their assembly into multisubunit complexes. Since their origin as a cyanobacterial endosymbiont, chloroplasts have retained downsized genomes and gene expression systems. However, most chloroplast proteins are encoded by the nuclear genome, synthesized in the cytosol, and then imported into the chloroplast. Therefore, the synthesis of thylakoid membrane protein complexes requires an intracellular coordination, which is mainly mediated via nucleus-encoded factors acting at all levels of chloroplast gene expression (for a recent overview, see [12]). Particularly chloroplast translation initiation has been considered to play a key role in determining the levels of photosynthesis-related proteins (for recent overviews, see [12]–[14]). Moreover, targeting of mRNAs to specific subcellular sites for localized translation contributes a further level of regulation of chloroplast gene expression [15],[16]. These targeting mechanisms are thought to involve membrane-associated RNA binding proteins (RBPs) that tether respective mRNAs to specific membrane regions within the chloroplast [16],[17].

The unicellular green alga *Chlamydomonas reinhardtii* is an established model organism for the analysis of biogenesis and gene expression in chloroplasts [18]. Moreover, it has the potential to be an ideal model system to study metabolic regulation of gene expression because it can adjust its metabolism to different energy sources. Unlike plants and animals, which derive energy exclusively from light and reduced carbon, respectively, *C. reinhardtii* can use both sources. It can exclusively use light energy in photoautotrophic growth, acetate in heterotrophic growth, or a combination of both in mixotrophic growth. The *C. reinhardtii psbA* mRNA is probably one of the most studied models of translational regulation in chloroplasts. The de novo assembly of PSII requires the synthesis of all subunits of this multimeric complex, including the *psbA* gene product, the D1 subunit, and its assembly partner D2. This thylakoid membrane protein synthesis is localized to a specialized membrane region called the T (translation)-zone surrounding the pyrenoid, a spherical body and the primary site of CO_2_ assimilation in the chloroplasts of most algae [19],[20]. However, under higher intensity light, D1 is damaged. Under these conditions, the so-called damage-repair cycle replaces degraded D1 proteins by newly synthesized ones (reviewed in [21]). Interestingly, repair synthesis of the PSII reaction center protein D1 is not localized to the T-zone but distributed over stroma-exposed thylakoid membranes indicating a strict spatial separation from the PSII de novo synthesis machinery [20].

Using in vitro RNA-binding assays, we previously identified an RBP of 63 kDa, RBP63, in *C. reinhardtii*, which is associated with stromal thylakoids and preferentially binds to an A-rich element within the 5′ UTR of the *psbA* mRNA [17]. This A-rich element is important for *psbA* translation [22]. Based on these findings, RBP63 was suggested to fulfill a role in membrane targeting and translation of the *psbA* mRNA [17].

Here, we report the identification and further characterization of RBP63, which surprisingly turned out to be the dihydrolipoamide acetyltransferase subunit, DLA2, of the cpPDC. Therefore, DLA2 might represent another example of a metabolic enzyme with an alternate function as an RBP involved in gene expression. Our data support a role of DLA2 in the localized translation of the *psbA* mRNA and confirm its known enzymatic role in acetyl Co-A synthesis. Moreover, the binding of this subunit to RNA might not be restricted to *C. reinhardtii* but appears to be a more general feature of dihydrolipoamide acetyltransferases, including the mitochondrial forms. Taken together, these results lead us to a new concept for the coordination of chloroplast translation and carbon metabolism that involves a cross-talk between protein and lipid synthesis in thylakoid membrane biogenesis.

## Results

### RBP63 Is DLA2, the Predicted E2 Subunit of cpPDC

**Figure 1:**
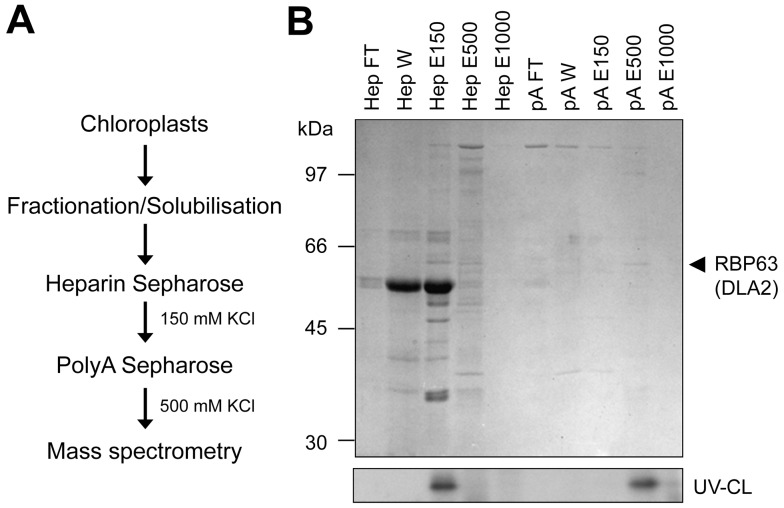
Isolation of RBP63. (A) Flow chart demonstrating the steps used to purify RBP63 (DLA2). (B) SDS-PAGE and Coomassie Blue staining of proteins at various stages of purification (upper panel) and UV cross-linking (UV-CL) of RBP63 to radiolabeled *psbA* 5′ UTR RNA (bottom panel). HepFT/HepW, flow through/wash fractions from heparin Sepharose; HepE 150, 500, and 1000, eluates obtained with 150, 500, and 1,000 mM KCl from heparin Sepharose, respectively; pA FT/W, flow-through/wash fraction from poly(A)-Sepharose column; pA E150, 550, and 1000, eluates obtained with 150, 500, and 1,000 mM KCl, respectively. The RBP63 protein that eluted with 500 mM KCl from poly(A)-Sepharose is marked by an arrow.

**Figure 2:**
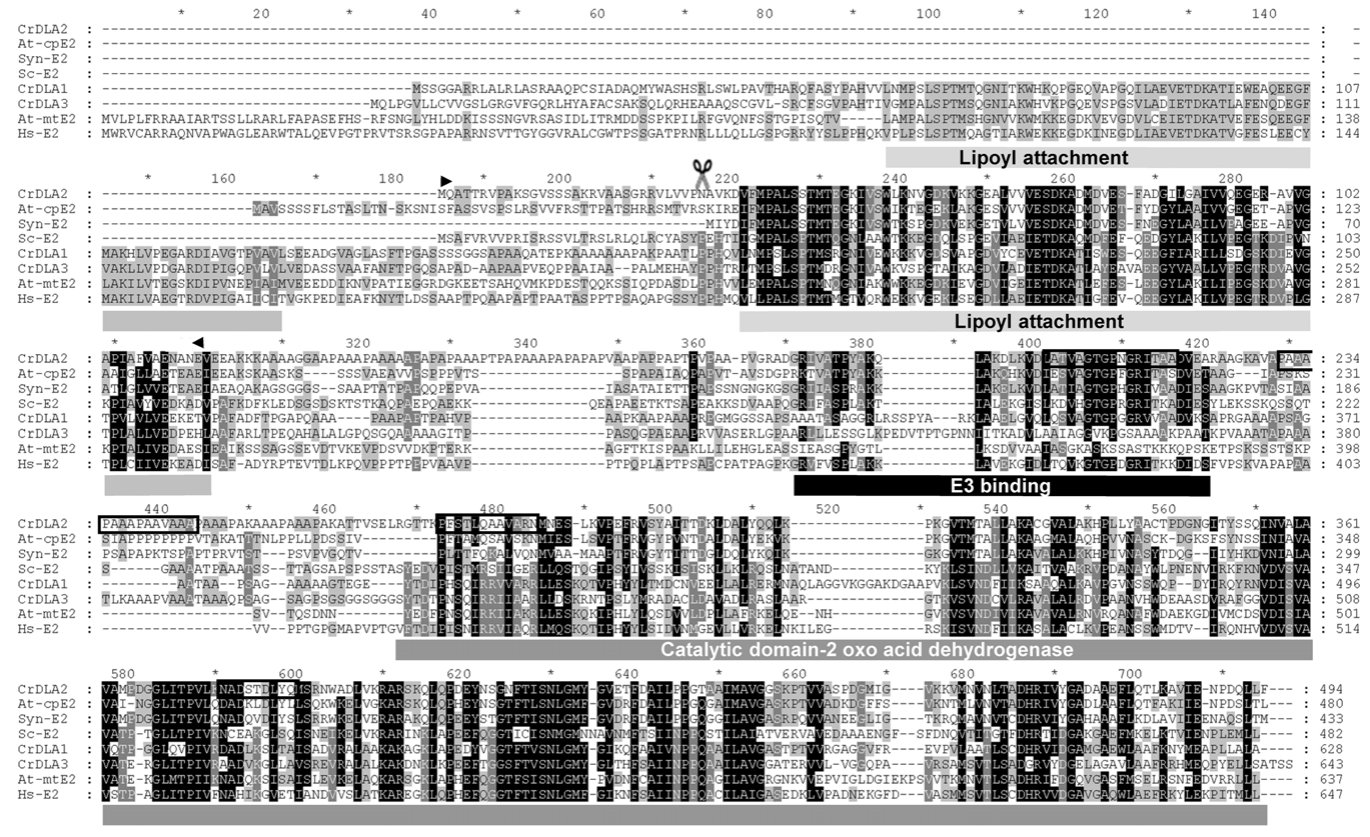
Three homologous dihydrolipoamide-acetyltransferases (DLAs) in *C. reinhardtii*. Multiple alignment of deduced amino acid sequences of the three DLA genes of *C. reinhardtii* along with *Synechocystis* sp. PCC 6803, human, *S. cerevisiae*, and *A. thaliana* sequences. Conserved residues are shaded in black, similar residues in grey. The sequences were aligned with ClustalW, manually edited and visualized using GeneDoc [81],[82]. The predicted chloroplast transit peptide of CrDLA2 has a length of 30 amino acids and is indicated by scissors. Functional domains are depicted below the sequences. Boxed regions in the DLA2 amino acid sequence correspond to peptides identified by mass spectrometry. Arrowheads indicate the amino acids fused to GFP (Figure 3B). Accession numbers are as follows: *C. reinhardtii* CrDLA1 (EDP01882), CrDLA3 (XP_001696403), CrDLA2 (model: au5.g10333 annotated in JGI v4; Joint Genome Institute; http://genome.jgi-psf.org/Chlre4/Chlre4.home.html); *A. thaliana* At-mtE2 (LTA3, AAV97810), At-cpE2 (LTA2, NP_189215); *Synechocystis* sp. 6803 Syn-E2 (sll1841, NP_441936); *H. sapiens* Hs-E2 (DLAT, NM_001931); and *S. cerevisiae* Sc-E2 (Lat1, NP_014328).

RBP63 was purified from detergent-solubilized thylakoid membranes of *C. reinhardtii* by two sequential affinity chromatography steps, using first heparin and then poly(A). RBP63 was monitored in the fractions on the basis of its *psbA* mRNA binding activity with a UV-cross-linking assay (Figure 1). We observed the highest RNA binding activity in the fraction eluted with 0.5 M KCl from poly(A)-Sepharose (Figure 1B, pA E500). SDS-PAGE and subsequent Coomassie Blue staining revealed that this fraction contained predominately the 63 kDa protein as well as minor amounts of proteins with different sizes (Figure 1B, pA E500). For the identification of the gene encoding RBP63, the excised 63 kDa protein species was then subjected to proteolytic digestion and subsequent analysis by mass spectrometry. Surprisingly, four different peptides mapped to DLA (DLA2), the E2 subunit of the cpPDC (Figure 2).

Blast searches of the *C. reinhardtii* nuclear genome using the DLA2 amino acid sequence identified two homologues: DLA1 and DLA3. However, the mass spectrometrically identified peptides unambiguously assigned the purified protein to DLA2 (Figure 2). Moreover, in silico analysis of the N-terminal region of DLA2 by TargetP [23] revealed properties of a 30 aa N-terminal chloroplast transit peptide, while DLA1 and DLA3 were predicted to localize to mitochondria, and thus are likely to represent putative E2 subunits of the mitochondrial PDC. Mitochondrial localization of DLA1 and DLA3 is further supported by their identification during mass spectrometrical determination of the *C. reinhardtii* mitochondrial proteome [24],[25].

To establish the gene structure of *DLA2*, we sequenced the EST clone MXL069g06 (BP097085) from the Kazusa DNA Research Institute. The clone included the full-length DLA2 cDNA, whose sequence corresponds to the gene model au5.g10333_t1 (chromosome 3:1419737–1423311) annotated in JGI v4 (Joint Genome Institute; http://genome.jgi-psf.org/Chlre4/Chlre4.home.html). The 2,174 nt *DLA2* transcript contains six exons, comprising 1,485 nt, flanked by untranslated regions of 38 and 651 nt at its 5′ and 3′ ends, respectively. In silico analysis of the genome sequence and DNA hybridizations confirm that *DLA2* is a single copy gene (Figure S1). The predicted protein has 494 amino acids with a molecular mass of 49.8 kDa. The discrepancy between the predicted molecular weight of DLA2 (49.8 kDa) and the apparent MW determined from SDS-PAGE (63 kDa, Figure S7) represents a size anomaly observed for all PDC E2 subunits analyzed to date and has been explained by frequently occurring turn-inducing and charged amino acid residues within the interdomain linker regions of these proteins [26]–[29]. Consistently, the same phenomenon was found with recombinant His-DLA2 protein (see below).

In agreement with α-proteobacterial and cyanobacterial origins of mitochondrial and chloroplast E2 subunits, respectively, DLA2 shows an overall amino acid sequence identity of more than 50% to the E2 subunit from the cyanobacterium *Synechocystis* sp. PCC 6803 and the chloroplast E2 orthologue from the vascular plant *Arabidopsis thaliana* (also named LTA2, At3g25860). In contrast, DLA2 shows only an identity of ∼30% to DLA1, DLA3, and mitochondrial orthologues from *A. thaliana*, *S. cerevisiae*, and *H. sapiens* [30]. The DLA2 sequence exhibits conserved regions for lipoamide attachment, E3 subunit binding, and a C-terminal 2-oxoacid dehydrogenase catalytic domain (Figure 2). The lipoamide attachment site and the catalytic domain show a relatively high conservation between the different organisms, whereas the E3 binding region is more variable. *C. reinhardtii* DLA2 contains a single predicted lipoamide attachment site, similar to the *Synechocystis* sp. PCC 6803 protein and the chloroplast E2 subunit from *A. thaliana*, whereas DLA1 and DLA3 exhibit two lipoyl binding sites similar to the mitochondrial enzymes from *A. thaliana* and *H. sapiens* (Figure 2).

### DLA2 Is a Chloroplast Localized Protein

**Figure 3:**
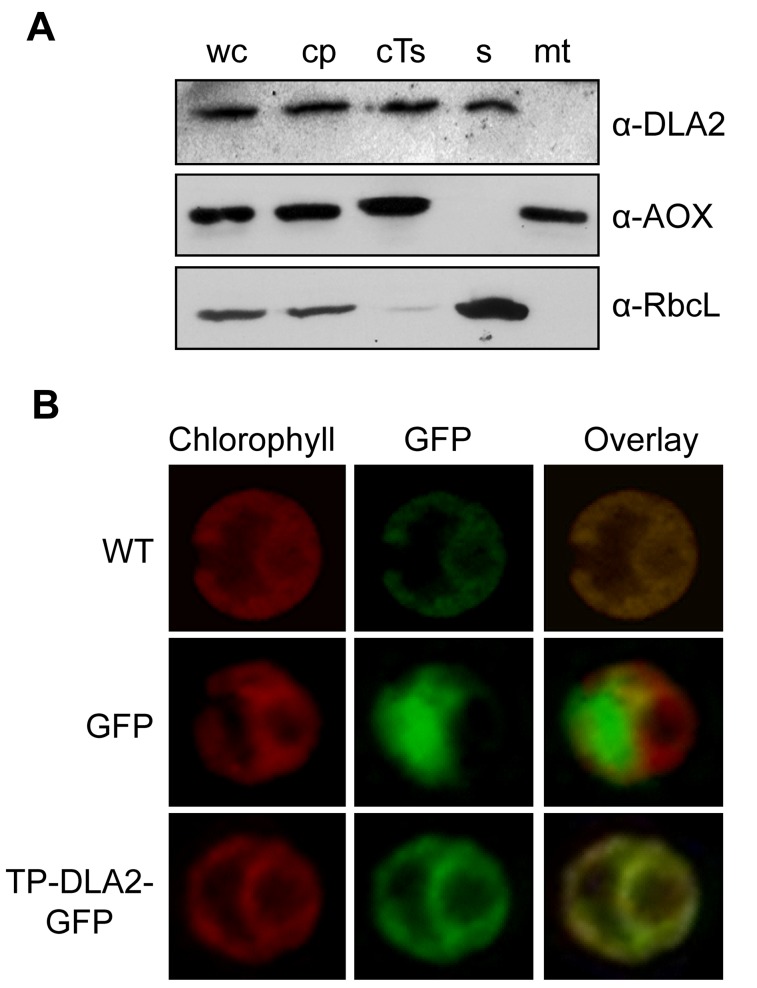
Plastid localization of DLA2. (A) Cell subfractionation. Cell fractions were prepared as described in Materials and Methods. We separated 30 µg of each protein fraction by SDS-PAGE and subjected them to immunoblot analysis. The blot was probed with antibodies against DLA2 (α-DLA2), the large subunit of Rubisco (α-RbcL), and the mitochondrial AOX (α-AOX, Agrisera). cp, chloroplasts; cTs, crude thylakoids; mt, mitochondria; s, stroma; wc, whole cells. (B) Analysis of the accumulation of DLA2–GFP fusion proteins in the transformed algal UVM4 strain by laser-scanning microscopy. Chlorophyll autofluorescence (Chlorophyll) and expression of GFP without (GFP) or with an N-terminal fusion of the first 114 aa of DLA2 (TP–DLA2–GFP). The untransformed UVM recipient strain served as control (WT). A merged image of the chlorophyll autofluorescence and GFP signals is shown (Overlay).

To verify the predicted chloroplast localization and exclude an additional targeting of DLA2 to mitochondria, we first carried out subcellular fractionation detecting DLA2 with an antiserum raised against the recombinant protein. The antibody detected a protein with an apparent molecular weight of 63 kDa in whole cell extracts (Figure S7). Subsequently, the comparisons of the level of DLA2 in subcellular fractions revealed it to be localized to chloroplasts, with approximately similar levels in thylakoid membranes versus the soluble stromal compartment (Figure 3A). As is often seen for *C. reinhardtii*, the chloroplast and thylakoid fractions were contaminated with mitochondria as judged by following the mitochondrial alternative oxidase (AOX; Figure 3A [31],[32]). However, the most significant result of this analysis was that no DLA2-specific signal was detected in the mitochondrial fraction, confirming that DLA2 does not form part of the mitochondrial PDC. In addition, this result reveals that a cross-reactivity of the antibody to the putative mitochondrial isoforms, DLA1 and DLA3, is improbable.

As a second independent approach to determine the intracellular localization(s) of DLA2, a DLA2–GFP fusion protein was expressed in *C. reinhardtii* (Figure 2). The DLA2–GFP signal was detected in the chloroplast, which was revealed by chlorophyll autofluorescence (Figure 3B). By contrast, when only GFP was expressed from the same expression vector, its signal was detected primarily in the central nuclear-cytosolic region.

### DLA2 Is in a High Molecular Weight (HMW) Complex with the *psbA* mRNA

**Figure 4:**
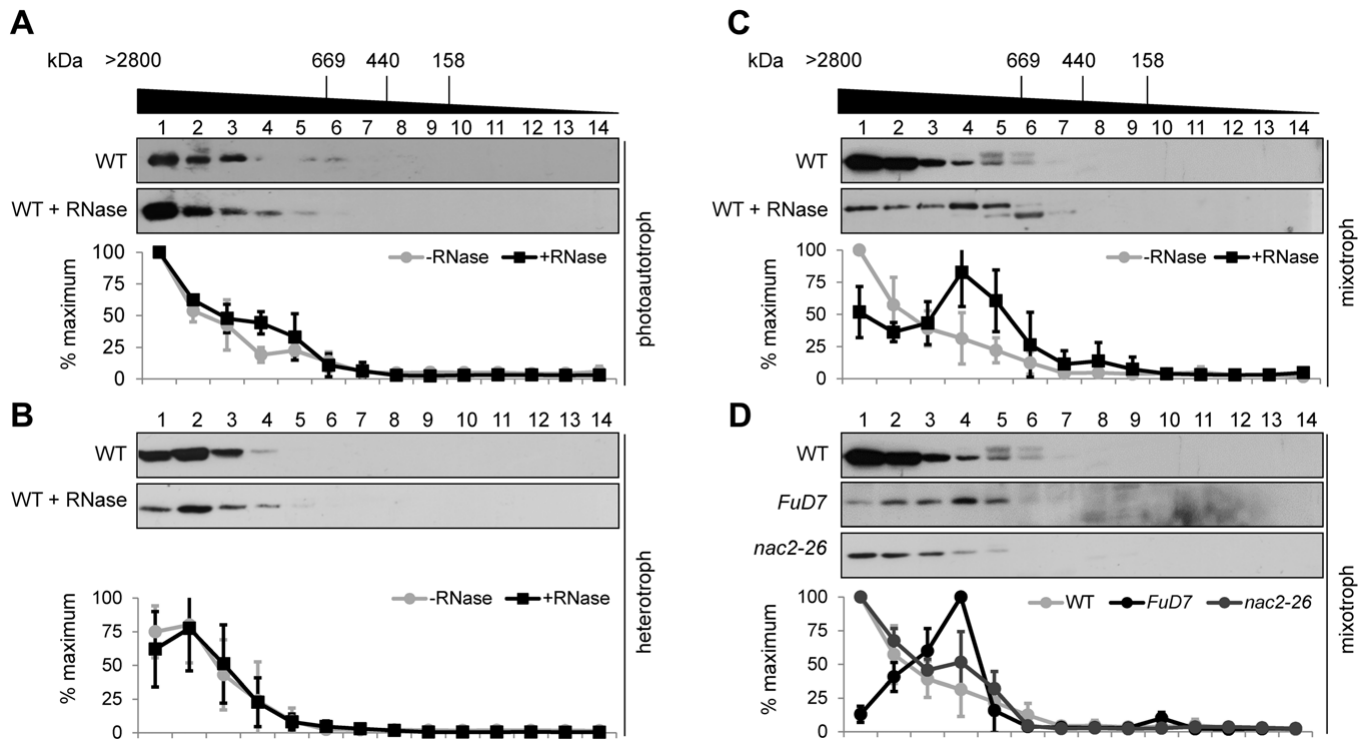
DLA2 forms part of a HMW complex that contains the *psbA* mRNA. SEC analyses of the DLA2 complex of wild-type CC-406 cells grown under photoautotrophic (A), heterotrophic (B), or mixotrophic (C) conditions along with PSII mutants *FuD7* and *nac2–26* grown under mixotrophic conditions. (D) Solubilized crude thylakoid proteins (treated with RNase or not) were separated by SEC. Fractions 1 to 14 were subjected to protein gel blot analyses using the DLA2 antiserum. Molecular masses shown at the top were estimated by parallel analysis of high molecular mass calibration markers. Below each panel, a quantitation of DLA2 signal intensities on Western blots for each condition is presented. The quantitation of signals was performed by using AlphaEaseFC software (Alpha Innotech Corp.). For each experiment, the highest amount of DLA2 in the SEC fractions was set to 100%. Mean values and error bars were calculated from at least three independent experiments.

Because DLA2 (RBP63) was previously characterized as a *psbA* mRNA binding protein, we tested whether DLA2 forms part of a HMW ribonucleoprotein (RNP) complex. Detergent-solubilized thylakoid membranes were prepared from wild-type cells cultured under photoautotrophic (light, no acetate), mixotrophic (light, with acetate), or heterotrophic (no light, with acetate) conditions and then subjected to size exclusion chromatography (SEC, [33]). By following the elution pattern of DLA2, we verified that it forms part of a HMW complex in a size range between 700 kDa and more than 2,800 kDa under each of the conditions (Figure 4). Peak fractions of eluted DLA2 slightly varied between samples from light versus dark grown cells. Under photoautotrophic and mixotrophic growth conditions, the DLA2 complex was more than 2,800 kDa, as indicated by a peak of elution in fraction 1 (Figure 4A,C). In cells grown heterotrophically, the peak fractions were moderately shifted toward smaller molecular sizes (i.e., fraction 2) (Figure 4B). This suggests a light-dependent formation of these different DLA2 HMW complexes. To investigate whether the observed HMW complexes contain RNA, solubilized thylakoid membranes were treated with RNase prior to SEC analysis. Intriguingly, the detected complexes revealed a growth condition-dependent RNase sensitivity: only under mixotrophic conditions did the complex shift toward lower molecular weight fractions upon RNase treatment (Figure 4C, peak of elution in fraction 4). In contrast, no such size shifts were observed for material from cells grown under photoautotrophic or heterotrophic growth conditions (Figure 4A,B). This strongly suggests that DLA2 forms a HMW–RNP complex in an acetate and light-dependent manner.

As localization studies revealed an almost equal distribution of DLA2 in the stroma and the membrane fraction (Figure 3A), we also investigated the size and RNase sensitivity of stromal DLA2 complexes of the wild-type grown under different conditions by SEC (Figure S2). In contrast to what was seen for the membrane-bound DLA2 complex (Figure 4A–C), we did not observe significant RNase sensitivity of stromal DLA2 complexes under any condition.

To obtain an indication of which of the gel filtration fractions contained active PDH complexes and additionally investigate if it is the active PDH complex itself that binds RNA or an alternative RNP complex, we performed cpPDC enzyme tests on the SEC fractions by measuring the reduction of NAD^+^ spectrophotometrically. These assays were performed on stroma or thylakoid SEC fractions from mixotrophically grown wild-type cells. The results revealed cpPDC activity mainly in the highest molecular weight fraction as expected (Figure S3). Approximately 10% of the activity measured in fraction 1 was detected in fraction 2 in a size range of ∼1.2–1.7 MDa, whereas no significant cpPDC activity was detectable in fractions containing smaller complexes. Moreover, no changes in the elution profile of cpPDC activity were observed upon RNase treatment even though significant amounts of DLA2 proteins were detected in lower molecular weight fractions 4 and 5. Therefore, it is probably not the active cpPDC that binds RNA (Figure S3A).

To test whether the RNA bound by the DLA2 complex is the *psbA* mRNA, solubilized thylakoid membranes from the chloroplast *psbA* deletion mutant *FuD7* (grown under mixotrophic conditions) were subjected to SEC (Figure 4D, [34]). With this mutant, the DLA2 complex was mainly detected in fraction 4, resembling the elution pattern of RNase-treated DLA2 complexes from mixotrophically grown wild-type cells. Reduced amounts of HMW complexes were detected in fractions 1+2 as compared to the WT, indicating that the lack of *psbA* mRNA in a mutant background reduces the DLA2 complex size. Therefore, this result supports the *psbA* mRNA as being the RNA component of the DLA2 RNP complex.

We cannot exclude DLA2 binding to RNAs other than the *psbA* message. However, another mixotrophically grown PSII mutant (*nac2–26*) tested in this analysis, which lacks the *psbD* mRNA, was not affected in DLA2 complex formation (Figure 4D [35],[36]). In conclusion, the data revealed a *psbA* mRNA-dependent DLA2 RNP complex under mixotrophic growth conditions.

To further verify a specific interaction of DLA2 and the *psbA* mRNA in vivo, RNA co-immunoprecipitations (co-IPs) using solubilized thylakoids from mixotrophically grown wild-type cells were performed (Figure S4). The immunoprecipitate obtained with the αDLA2 antiserum contained the *psbA* mRNA, but not the mRNAs of *rbcL* or *atpB*, chloroplast genes encoding the large subunit of Rubisco and the beta subunit of ATP synthase, respectively. This confirms the formation of a specific *psbA* mRNA/DLA2 complex in vivo (Figure S4B). In agreement with the results of SEC analysis, no *psbA* mRNA was co-immunoprecipitated from cells grown under heterotrophic conditions (Figures 4B and S4B). It should be noted that this IP was relatively inefficient because the DLA2 antiserum only weakly recognizes the native protein (Figure S4A).

**Figure 5:**
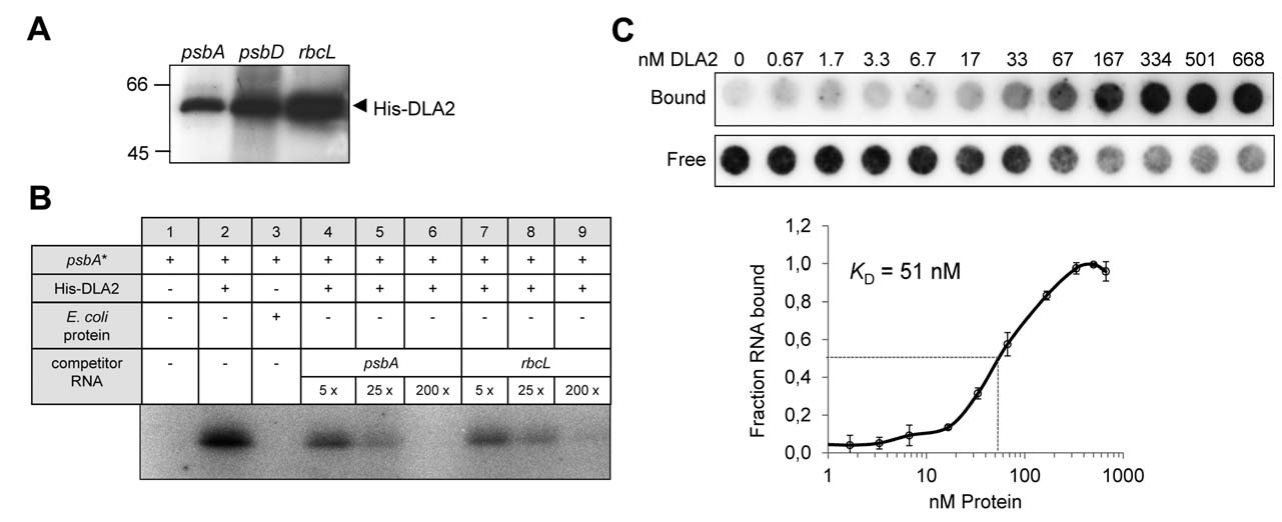
Intrinsic RNA binding activity of recombinant His-DLA2 protein. (A) UV cross-linking experiment. A total of 200 ng of purified His-DLA2 were analyzed by UV cross-linking using 50–100 kcpm of radiolabeled *psbA*-, *psbD*, or *rbcL*- 5′ UTR RNA probes. (B) Competition experiments. We incubated 10 ng of His-DLA2 protein with radiolabeled *psbA*-RNA (psbA*) and a 5-, 25-, or 200-fold molar excess of the indicated competitor RNAs representing the 5′ UTRs of the *psbA* or *rbcL* mRNA, respectively. To exclude an unspecific RNA binding of contaminating *E. coli* proteins, we used the same volumes as used for the DLA2 protein of an elution fraction obtained from the bacterial host strain transformed with the empty expression vector served as control (*E. coli* protein, lane 3). (C) Equilibrium binding constant (*K*_D_) of recombinant DLA2 protein. Binding reactions containing 6.7 pM ^32^P-labeled *psbA* 5′ UTR RNA and indicated molarities of DLA2 were filtered through stacked nitrocellulose and nylon membranes (upper panel) using a dot blot apparatus according to Ostersetzer et al. [38]. Signal intensities of nitrocellulose-bound protein–RNA complexes (Bound) as well as nylon membrane-bound free RNAs (Free) were quantified. The *K*_D_ value was determined from three experiments performed with the same DLA2 preparation (lower panel).

As a next step, we further substantiated the RNA binding capacity of DLA2 by testing the hexahistidine-tagged recombinant protein (His-DLA2) for in vitro RNA binding activity (Figure 5). In UV cross-linking experiments with various ^32^P-labeled RNAs, His-DLA2 showed a clear intrinsic RNA binding activity for all probes tested (e.g., the 5′ UTRs of *psbA*, *psbD*—encoding the PSII reaction center protein D2—and *rbcL*) (Figure 5A). We next tested the specificity of RNA binding by applying RNA competition assays. Therefore, the UV cross-linking assays were performed in the presence of increasing concentrations of homologous or heterologous unlabeled RNA probes as competitors (Figure 5B). In contrast to what was previously reported for the native DLA2 protein, His-DLA2 exhibited no specificity for the *psbA* RNA ([17], Figure 5B). This is probably due to a lack of other components of the DLA2–RNP complex that facilitate recognition of the *psbA* 5′ UTR by DLA2 in vivo. Such a behavior would resemble other chloroplast RBPs, like RBP40 from *C. reinhardtii*, which—as an isolated protein—unspecifically recognizes any RNA. Only as part of a complex with its cognate binding partner Nac2 does RBP40 have binding specificity to the *psbD* mRNA [37].

However, even though the protein appeared to bind nonspecifically to the *psbA* mRNA in vitro, we determined its equilibrium binding constant (*K_D_*) for the *psbA* 5′ UTR to put DLA2 on a comparable basis with other known RBPs (Figure 5C). To this end, we applied an independent filter binding assay according to Ostersetzer et al. [38], which does not involve a UV cross-linking step. The obtained *K_D_* value of ≈51 nM is in line with those determined for other RBPs (for examples, see [38]–[40]).

### DLA2 Is Involved in D1 Protein Synthesis

To further elucidate the role of DLA2 in *psbA* expression and acetyl-CoA production, *DLA2*–RNAi lines were generated [41]. Of ca. 800 transformants, ca. 100 clones survived the selection procedure. Three of these lines, namely *iDLA2*-1, -2, and -3, exhibited the highest reductions of DLA2 level, which were determined to be 5% (±2), 10% (±4), and 6% (±2)%, respectively, relative to the DLA2 level in the recipient strain transformed with the empty vector. These strains maintained the DLA2 deficiency for over 2 years under selection for the RNAi-induced phenotype.

**Figure 6:**
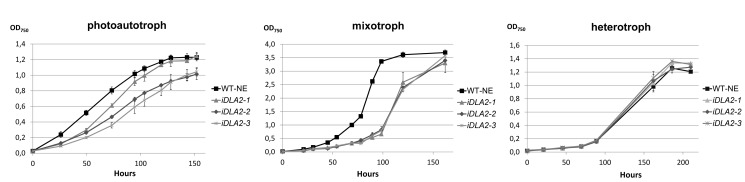
Growth curves of *iDLA2* lines. Growth rates were determined for the wild-type strain UVM4 transformed with the empty vector NE537 (WT-NE) and *iDLA2* lines (*iDLA2*-1, -2, -3) under photoautotrophic, mixotrophic, or heterotrophic growth conditions by measuring OD_750_. Error bars represent 1 standard deviation from the mean based on results from three independent cultures.

To explore the phenotypic consequences of *DLA2* deficiency in these RNAi lines, their growth rates were monitored under photoautotrophic, mixotrophic, and heterotrophic conditions (Figure 6). Growth of RNAi lines was only slightly affected under photoautotrophic and not at all affected under heterotrophic conditions. However, under mixotrophic conditions, severe growth retardation was observed for the *DLA2* knock-down strains as compared to the wild-type. Therefore, DLA2 seems to be required for wild-type growth rate specifically under the condition associated with the formation of an RNP complex (Figure 4).

**Figure 7:**
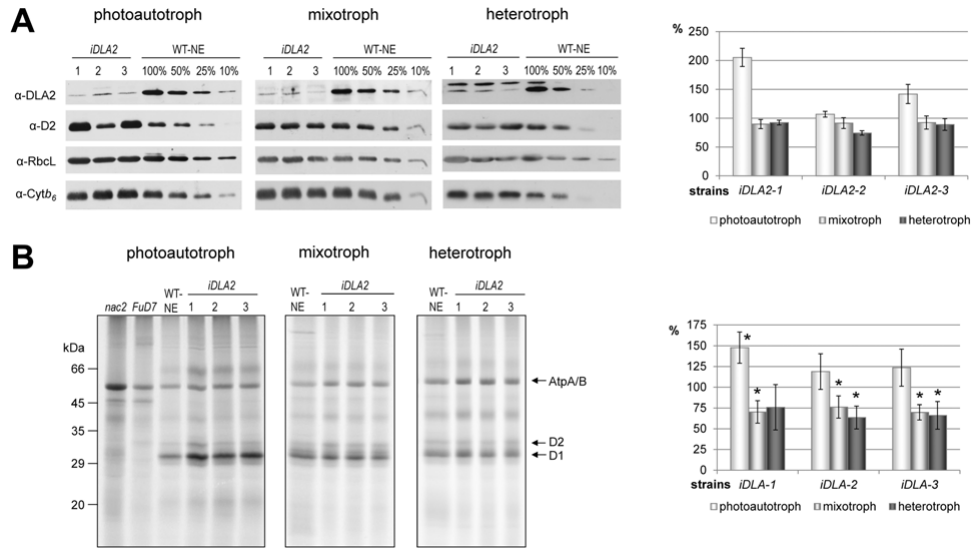
D1 accumulation and synthesis in *iDLA2* lines under different growth conditions. (A) D1 protein accumulation. Immunoblot analysis of 40 µg of total cell proteins from indicated *iDLA2* lines along with a dilution series of the control strain transformed with the empty vector (WT-NE). Cells were cultured photoautotrophically, mixotrophically, or heterotrophically. Protein gel blot analyses were performed using antibodies raised against the proteins indicated at the left (RbcL, large subunit of Rubisco; Cyt*b_6_*, cytochrome *b_6_*; D2, reaction center protein of PSII). Right panel demonstrates the quantification of immunoblot signals of three independent experiments. Quantification was performed using the AlphaEaseFC software (Alpha Innotech Corp.) by calculating the ratio of D2 over Cyt*b_6_*. Values obtained for WT-NE were set to 100%. Mean values and standard deviations from three independent experiments are shown. (B) D1 protein synthesis. Membrane proteins of depicted strains were pulse-labeled with ^35^S sulphate, fractionated in SDS-urea-gels, and analyzed by autoradiography. (Right panel) Quantification of signals was performed by using AlphaEaseFC software (Alpha Innotech Corp.) and calculating the ratio of D1 over AtpA/B. Values obtained for WT-NE were set to 100%. Growth conditions as indicated. Mean values and standard deviations from three (photoautotrophic) or five (mixotrophic/heterotrophic) independent experiments are shown. An asterisk indicates that a mean D1/AtpA/B labeling ratio is significantly different from that of WT-NE (100%) as determined by a one-sample *t* test (*p*<0.05).

To determine whether DLA2 is required for *psbA* expression and accumulation of PSII, D2 protein levels were measured in the *DLA2*–RNAi lines by immunoblot analyses. It was previously shown in *C. reinhardtii* that D1 and D2 accumulate in a 1∶1 stoichiometry, whereas unassembled proteins are rapidly degraded [42]–[44]. Therefore, D2 can be used as a proxy measure of D1 accumulation. Interestingly, we observed a light- and acetate-dependent accumulation of PSII in the *DLA2*–RNAi lines (Figure 7A). In contrast, under photoautotrophic growth conditions, PSII accumulation was higher in the RNAi lines as compared to the wild-type. In contrast, in lines cultured mixotrophically under the same light conditions or heterotrophically in the dark, RNAi-mediated DLA2 deficiency leads to slightly reduced PSII levels. Other protein complexes in the chloroplast were not affected in the RNAi lines, as judged by parallel monitoring of steady-state levels of the large subunit of Rubisco and cytochrome *b_6_* (Figure 7A).

To test whether altered *psbA* mRNA levels or D1 protein synthesis rates are responsible for the observed changes of the levels of D1 protein and, consequently, PSII accumulation, we performed Northern blots as well as ^35^S protein pulse labeling assays (Figure S5, Figure 7B). No alterations in *psbA* transcript levels were observed in *iDLA2* lines as compared to the wild-type from cells grown under photoautotrophic, mixotrophic, or heterotrophic growth conditions (Figure S5). However, whereas only minor effects on D1 protein accumulation were observed, D1 synthesis rates were clearly altered in the *iDLA2* lines relative to in the wild-type strain (Figure 7). For example, higher D1 protein synthesis rates in the *iDLA2* lines were detected under phototautotrophic conditions, whereas D1 synthesis rates were reduced under mixo- and heterotrophic growth conditions, respectively (Figure 7B). The less obvious effect of DLA2 deficiency on D1 protein accumulation under mixotrophic and heterotrophic conditions as compared to D1 protein synthesis might be explained by posttranslational stabilization effects counteracting a reduced translation rate.

### DLA2 Is Involved in *psbA* mRNA Localization

**Figure 8:**
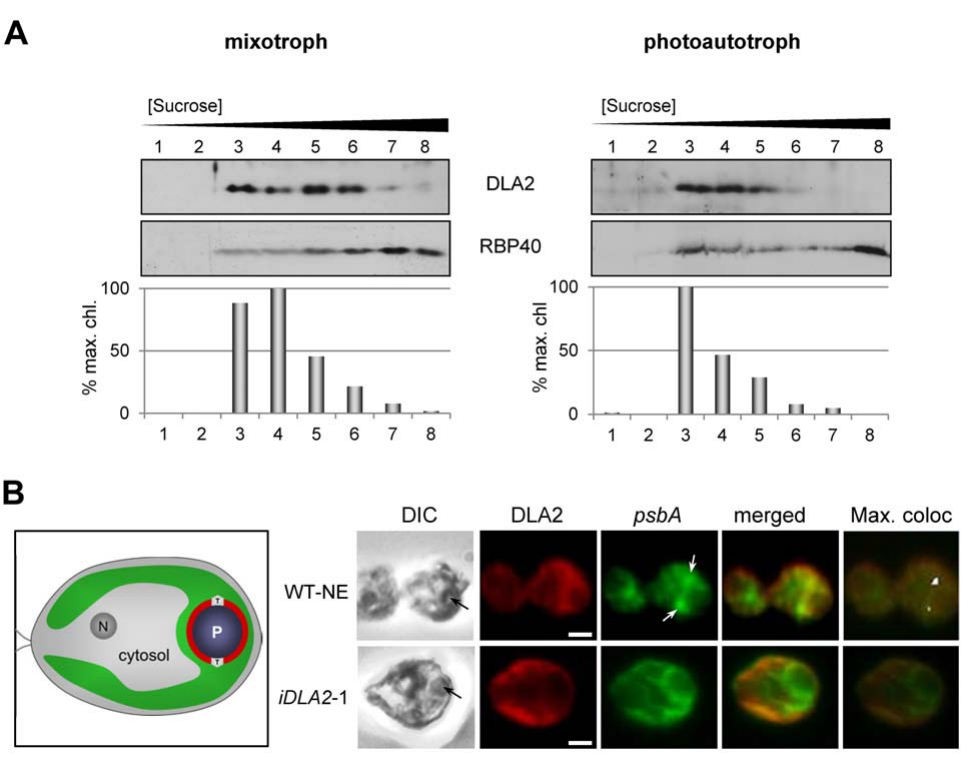
DLA2-dependent localization of the *psbA* mRNA to the PSII biogenesis region of the chloroplast (T-zone) and CTMs. (A) Membranes from cells from either mixotrophic (left panel) or photoautotrophic (right panel) conditions were fractionated by isopycnic sucrose gradient ultracentrifugation, and fractions were analyzed by immunoblot for DLA2 and a marker protein for CTM (RBP40). As a marker for thylakoid membranes, chlorophyll concentration is graphed as the percentage of the maximum value. (B) Cells of either the control strain transformed with the empty vector (WT-NE) or DLA2-deficient (*iDLA2*-1) were grown under mixotrophic conditions, IF-stained with the α-DLA2-antiserum (red), and FISH-probed for the *psbA* mRNA (green). Black arrows in differential interference contrast (DIC) images at the left indicate the position of the pyrenoid. White arrows indicate where the *psbA* FISH signal is localized in T-zones. Pixels with the strongest signals obtained by the program Colocalization Finder (ImageJ) were labeled white (max. confocal). Bars, 1 µm. An illustration of a *C. reinhardtii* cell shows the nucleus (N), cytosol, and chloroplast (green) with the T-zone (T) and pyrenoid (P).

As another means of testing whether DLA2 plays a role in *psbA* translation, we asked whether it is associated with a “chloroplast translation membrane” (CTM). This membrane subfraction was identified as a privileged location of translation in the *C. reinhardtii* chloroplast. CTMs are characterized by their higher density as compared to thylakoids and their association with the translation machinery including the translational regulator RBP40 [45]. Accordingly, membranes from wild-type cells from mixotrophic conditions were separated on the basis of density by floatation from a 2.5 M sucrose cushion into a 0.5–2.2 M sucrose gradient during isopycnic ultracentrifugation. Most thylakoid membranes were detected in the intermediate-density fractions 3 and 4, as revealed by their high chlorophyll concentrations (Figure 8A, left panel). Progressively less thylakoid membrane was present in fractions with increasing density (fractions 5–8). The CTM marker RBP40 was detected in fractions 3–8, as reported previously [45]. Immunodetection of DLA2 revealed that its concentrations were highest in lanes 3–6 and progressively decreased in the denser fractions 7–8. The presence of DLA2 in fractions 3 and 4 was inconclusive because this is consistent with DLA2 association with thylakoid membranes, CTM, or both membrane types. However, DLA2's sustained high levels in lanes 5 and 6 (i.e., in fractions in which thylakoid membrane levels progressively decrease) indicate that DLA2 is associated with a nonthylakoid membrane such as the CTM in these fractions (Figure 8A, left panel). That the RBP40 distribution extends to the bottom of the gradient (lanes 7 and 8) indicates that the DLA2 is not associated with the densest CTMs to detectable extent.

To determine whether the DLA2 that cofractionates with CTM (lanes 5 and 6) in membranes from mixotrophically cultured cells (Figure 8A, left panel) could be relevant to its role in *psbA* translation, we carried out the same analysis on membranes from cells cultured photoautotrophically, which lack the DLA2 RNP complex (Figure 4A). In this experiment, DLA2 more closely co-fractionated with the thylakoid membranes (Figure 8A, right panel, fractions 3–5). The density distribution of CTM was not dramatically altered relative to mixotrophically cultured cells, based on the distribution of RBP40. Together, these results substantiate the proposed acetate-dependent role of DLA2 in *psbA* translation.

It was proposed previously that DLA2 binds the *psbA* mRNA to localize it for translation and cotranslational membrane insertion of D1 [17]. To test this possibility, we asked whether DLA2 is required to localize the *psbA* mRNA to a specific, spatially defined, “translation zone” (T-zone) in the *C. reinhardtii* chloroplast, which is believed to be a privileged subcompartment for protein synthesis and to contain the CTM [16],[20]. The T-zone was originally defined by results of confocal microscopy, which revealed in the outer perimeter of the pyrenoid by the colocalization of the *psbA* mRNA, chloroplast ribosome subunits, and the PSII translation factor RBP40 (RB38) [20]. The pyrenoid serves as a cytological landmark for the T-zone because it is large (1–2 µm in diameter) and located in the same position in every chloroplast (Figure 8B). To determine whether DLA2 localizes the *psbA* mRNA to the T-zone, we first asked whether this localization is altered in the most severe RNAi line: *iDLA2*-1. When we visualized the suborganellar distribution the *psbA* mRNA by fluorescence in situ hybridization (FISH) in the recipient strain used for RNAi, WT-NE, we found that the *psbA* mRNA was localized to the T-zone in 78% of cells (Figure 8B). In the representative WT-NE cell, the green *psbA* FISH signal is concentrated in distinct regions adjacent to the pyrenoid, which is seen in the accompanying phase contrast image. These cells were from the mixotrophic growth conditions in which DLA2/*psbA* mRNA complex formation was detected (Figure 4C). In contrast, when *iDLA2*-1 cells were examined, only 30% showed this *psbA* mRNA localization pattern, a greater than 2-fold reduction relative to WT-NE. In the representative *iDLA2-1* cell, the *psbA* FISH signal is more dispersed and not concentrated in distinct regions adjacent to the pyrenoid (Figure 8B). Together, these results reveal that DLA2 is involved in *psbA* mRNA localization to the T-zone and that the sustained *psbA* localization in 30% of these cells probably reflects the residual DLA2 in this RNAi line (i.e., 5% of the wild-type level, Figure 7A), the activity of a partially redundant DLA2-independent localization mechanism, or both.

As another means of determining whether DLA2 localizes the *psbA* mRNA, we asked whether they colocalize in the T-zone. When the WT-NE cells were immunofluorescence (IF) stained for DLA2, 65% showed the DLA2 IF signal near the pyrenoid (Figure 8B). However, these analyses were hampered by the dispersed IF signal, reflecting the difficulty of specifically detecting the RNA-binding form of DLA2 amidst the “background” IF signal from DLA2 of cpPDC, which is most probably nonlocalized based on its detection as soluble and membrane-bound forms (Figures 3A and S3). To reveal a pool of DLA2 involved in *psbA* mRNA localization, amidst the nonlocalized signal, we used the program Colocalization Finder (ImageJ) to display signals of maximal intensity from both DLA2 and the *psbA* mRNA [20]. Our prediction was that the strongest signals from each should be colocalized for translation. As shown in Figure 8B, clusters of maximal colocalized signals from DLA2 and the *psbA* mRNA were seen in the T-zone in 60% of the cells analyzed (and are labeled white in the right-hand most image). Moreover, this pattern requires DLA2 because parallel analyses of the most severe knock-down line, *iDLA2*-1, revealed that only 8% of these cells showed the colocalization with DLA2 in the T-zone (Figure 8B). Therefore, these in situ results and the biochemical evidence that DLA2 is associated with the CTM (Figure 8A) support our hypothesis that DLA2 is required for the localization of the *psbA* mRNA to the T-zone and, thereby, targets newly synthesized D1 protein to this PSII biogenesis center.

### DLA2 Is an Enzymatically Active Subunit of the cpPDC

**Figure 9:**
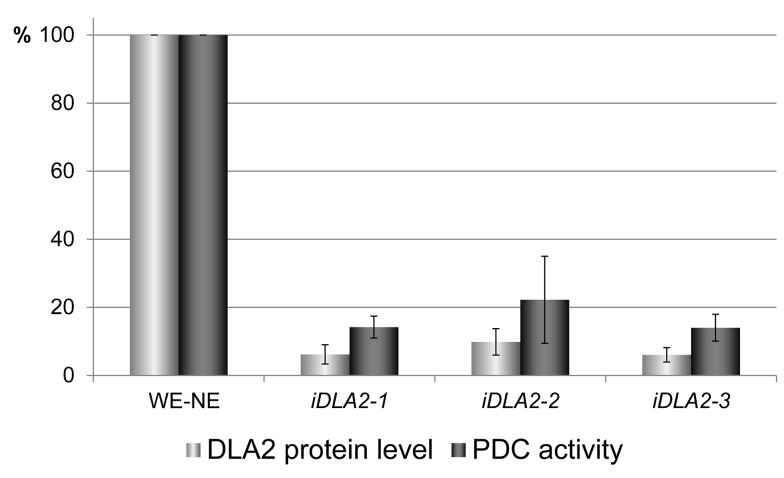
DLA2 is an active subunit of the chloroplast pyruvate dehydrogenase. cpPDC activity tests in the same *iDLA2* lines grown under photoautotrophic conditions were performed as described in Materials and Methods. The cpPDC activity measured in the wild-type transformed with the empty vector (WT-NE) is designated as 100%. Mean values and standard deviations from three independent experiments are shown. For comparison, DLA2 protein accumulation in *iDLA2* lines is shown aside (see also Figure 7A).

cpPDC catalyzes the oxidative decarboxylation of pyruvate to acetyl-CoA for chloroplast fatty acid synthesis with the concomitant generation of NADH [9],[11]. Thus, to test whether DLA2 is an active subunit of the cpPDC, enzyme activity assays were performed on protein extracts of photoautotrophically grown *DLA2*–RNAi lines by measuring the reduction of NAD^+^. As shown in Figure 9, cpPDC activity in extracts of the three *DLA2–*RNAi lines was reduced to approximately 15%–25% of the enzyme activity measured with the wild-type used for RNAi. The level of cpPDC activity thus correlated with the level of DLA2 protein in the RNAi lines (compare Figure 7A and Figure 9). We therefore conclude that DLA2 represents an active component of the chloroplast PDH complex. However, reduced enzyme activity did not lead to dramatic changes in overall lipid accumulation as assayed by thin layer chromatography (Figure S6). This might be explained by sufficiently high residual cpPDC levels in the investigated RNAi lines that still mediate efficient production of acetyl-CoA for fatty acid synthesis.

### Regulation of cpPDC Activity

In order to explore possible signals for the transition of the enzymatic form of DLA2 to its RNA-binding form for *psbA* translation, we asked whether cpPDC activity is altered by interaction of DLA2 with the *psbA* mRNA. This idea is supported by bioinformatical analysis of the DLA2 amino acid sequence. By using the RNABindR software to predict possible RNA-binding residues within the amino acid sequence of DLA2 [46], we detected a putative RNA-binding region that overlaps the E3 subunit attachment site (aa 191–216, compare Figure 2 and Figure S9A). Hence, a competitive binding of either E3 or *psbA* mRNA to this DLA2 site might be involved in regulation. Since binding of E3 is required for cpPDC function, the lack of *psbA* mRNA should then lead to increased cpPDC activity. We therefore decided to analyze cpPDC activity in the absence of *psbA* mRNA.

**Figure 10:**
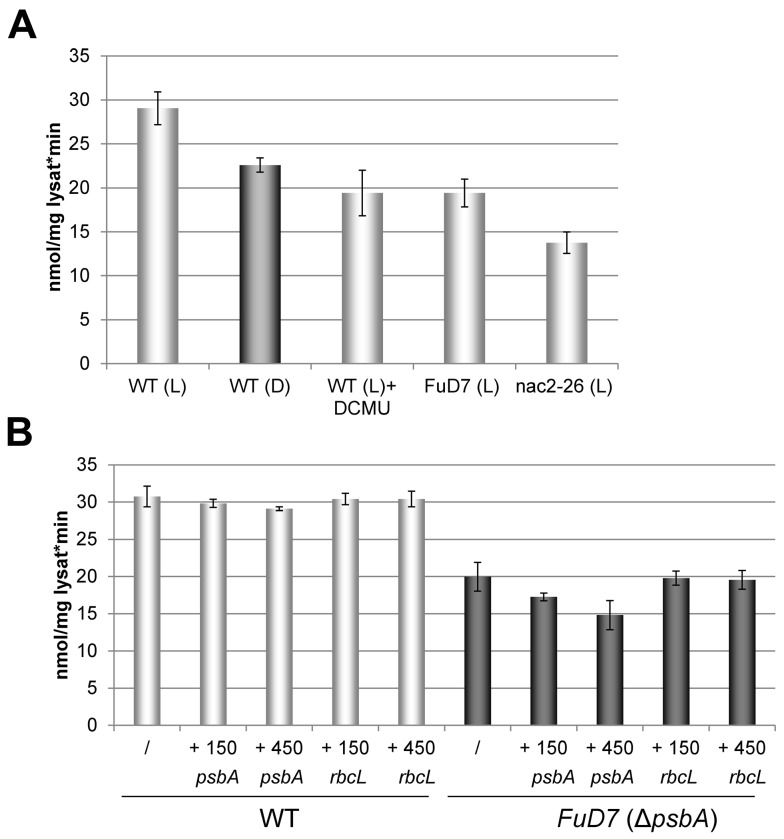
Regulation of cpPDC activity. (A) Regulation of the cpPDC by photosynthetic electron flow. cpPDC activity in cell-wall-deficient wild-type cells (WT) and PSII mutants *FuD7* and *nac2–26* grown in the presence of acetate under moderate light conditions (L) or in the dark (D). If indicated, wild-type cells were treated for 3 h with 20 µM DCMU (WT + DCMU). Activity is expressed as nmole NADH formed/min and mg protein. The mean values and standard deviations from three independent measurements are shown. (B) Regulation of cpPDC activity by *psbA* mRNA. cpPDC activity was measured in cell-wall-deficient wild-type cells (WT) and in the *FuD7* mutant. If indicated, lysates were incubated prior to cpPDC activity measurement for 10 min at room temperature with 150 pmol or 450 pmol of RNA derived from *psbA* or *rbcL* 5′ UTRs, respectively. Activity is expressed as nmole NADH formed/min and mg protein. The mean values and standard deviations from three independent measurements are shown.

Data from higher plants have revealed an activation of the complex by light, due to an increase of stromal pH and Mg^2+^ concentration (reviewed in [11]). A similar light-dependent activation of cpPDC could be confirmed for *C. reinhardtii* wild-type as indicated by an increase of activity of ca. 30% of light versus dark grown cells (Figure 10A). This light activation depends on electron transport from PSII to PSI, as indicated by clearly reduced cpPDC activity upon treatment of cells with DCMU, a chemical that specifically blocks the electron flow from PSII (Figure 10A). Next, we tested cpPDC activity in the PSII mutants *FuD7* and *nac2–26* specifically lacking either the *psbA* or the *psbD* mRNA, respectively. Consistent with a requirement for photosynthetic electron flow, both mutants exhibited reduced cpPDC activities as compared to the wild-type. However, in the *FuD7* mutant, this reduction (66% of wild-type level) was less pronounced than in *nac2-26* (47% of wild-type level; Figure 10A). This might suggest that the *psbA* mRNA, which is still present in *nac2–26* but lacking in *FuD7*, has an additional inhibitory effect on cpPDC activity.

To further test this, we measured the cpPDC activity in extracts of light grown wild-type and *FuD7* cells after incubation with in vitro transcribed RNAs derived from the 5′ UTRs of the *psbA* or *rbcL* mRNAs (Figure 10B). Whereas we detected only minor changes of cpPDC activity in the wild-type upon addition of exogenous *psbA* or *rbcL* RNAs, a dose-dependent reduction of enzyme activity was obtained for the *FuD7* mutant after incubation with *psbA*, but not with *rbcL* RNA. Addition of 450 pmol *psbA* RNA thereby significantly (*p*<0.05) reduced the activity of *FuD7* cpPDC to approximately 48% of the activity measured in the wild-type. Therefore, the activity level measured for *nac2–26* strongly resembles that measured for *FuD7* in the presence of 450 pmol RNA, suggesting that the observed differences between *nac2–26* and *FuD7* are due to the absence of *psbA* mRNA in *FuD7* (Figure 10A,B). The marginal reduction of cpPDC activity upon addition of *psbA* mRNA to wild-type extracts might indicate a saturation of DLA2 with *psbA* mRNA under these conditions (Figure 10B). Taken together, our data strongly suggest that cpPDC activity is specifically affected by the presence of *psbA* mRNA, presumably via its binding to the E3 attachment site of DLA2.

### Is the Binding of RNA an Ancient Feature of dihydrolipoamide acetyltransferases?

**Figure 11:**
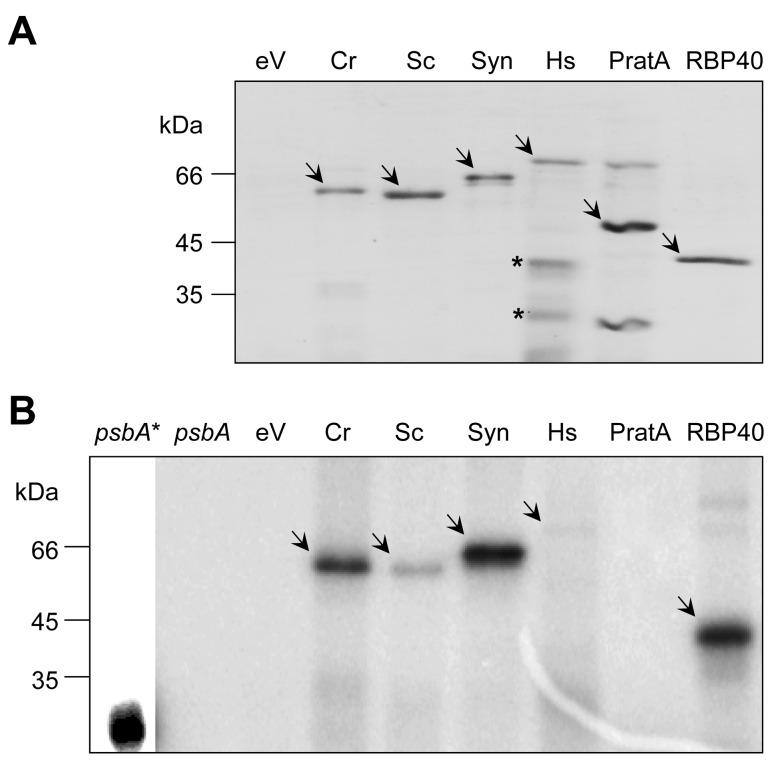
Binding of RNA by dihydrolipoamide acetyltransferases might be a global phenomenon. (A) Hexahistidine-tagged E2 fusion proteins from *C. reinhardtii* (Cr), *S. cerevisiae* (Sc), *Synechocystis* sp. 6803 (Syn), and *H. sapiens* (Hs) along with two control proteins (PratA and RBP40) were purified on Ni-NTA Sepharose, separated by SDS-PAGE, and Coomassie-stained. To exclude an unspecific RNA binding of contaminating *E. coli* proteins in (B), we used the same volumes as used for the *C. reinhardtii* E2 protein of an elution fraction obtained from the bacterial host strain transformed with the empty expression vector served as control (eV). Recombinant proteins are indicated by arrows. Proteins in the preparation of the human E2 subunit (Hs) that are specifically recognized by an anti-histidine antibody are marked by an asterisk. (B) RNA binding assay. One of the 20 (∼100 ng) recombinant proteins shown in (A) was used for UV cross-linking to *psbA* mRNA. Lanes “psbA*” and “*psbA*” show the radiolabeled *psbA* RNA without and with RNase treatment, respectively. Due to the high intensity of the psbA* signal, a lower exposure of this lane is shown. Specific radioactive signals are indicated by arrows.

The dihydrolipoamide acetyltransferase component of the PDC represents an evolutionary and functionally conserved gene family from prokaryotes to eukaryotes [9],[30],[47]. But does this also hold for its ability to bind RNA and fulfill a possible role in gene expression? To gain first insights, we predicted the possible RNA binding sites within the amino acid sequences of E2 subunits from phylogenetically distant organisms (i.e., human, yeast, and a cyanobacterium) (Figure S9). Similar to *C. reinhardtii* DLA2, all analyzed proteins revealed a putative RNA binding domain within the proposed E3 attachment site. Consequently, we tested recombinant E2 versions from these organisms for their ability to bind RNA in vitro. Therefore, E2 subunits were heterologously expressed in *Escherichia coli* as hexahistidine-tagged fusion proteins and purified on Ni-NTA Sepharose (Figure 11A). Even though the recombinant proteins were markedly enriched after purification, especially the preparation of the E2 subunit from human (Hs-E2) revealed contaminations with proteins in a size range of 30–45 kDa. These most likely represent Hs-E2 degradation products because they are specifically recognized by an anti-hexahistidine antibody (unpublished data, Figure 11A). As controls for the subsequent RNA binding assay, we also included PratA, which is an unrelated his-tagged protein not described to possess any RNA binding activity [48]. As a positive control, we used the RNA binding protein RBP40, which was already shown to unspecifically bind to RNA in vitro [49],[50]. Since no specific RNA targets of the different E2 subunits are known, *psbA* 5′ UTR RNA was used to detect general binding activity (Figure 11B). Interestingly, all tested E2 proteins showed a binding to the RNA probe applied in the UV cross-linking assay, suggesting that RNA binding is an intrinsic capacity of all E2 subunits, even of those from mitochondria and prokaryotes. However, the binding of the mitochondrial proteins, especially of the human protein, to the *psbA* RNA appeared to be weaker as compared to the recombinant proteins of the green lineage.

## Discussion

Our finding that RBP63 is DLA2 reveals another example of a metabolic enzyme with a dual function as an RBP. Some of these proteins, including enzymes involved in carbon and fatty acid metabolism, were shown to directly interact with nucleic acids and have been proposed to influence transcription or translation (reviewed in [4],[5]). However, the physiological significance of RNA binding by metabolic proteins is mostly unclear and might be a remnant of the “RNA-world” [51]. In this context, our results are important because they reveal a physiological function of an alternate RNA-binding form of a metabolic enzyme: that is, DLA2 as an RBP in *psbA* translation. Our results also suggest a model in which DLA2 adjusts *psbA* expression in response to available sources of energy.

### DLA2 Is an Active Subunit of cpPDC and Forms Part of an RNP Complex with the *psbA* mRNA

**Figure 12:**
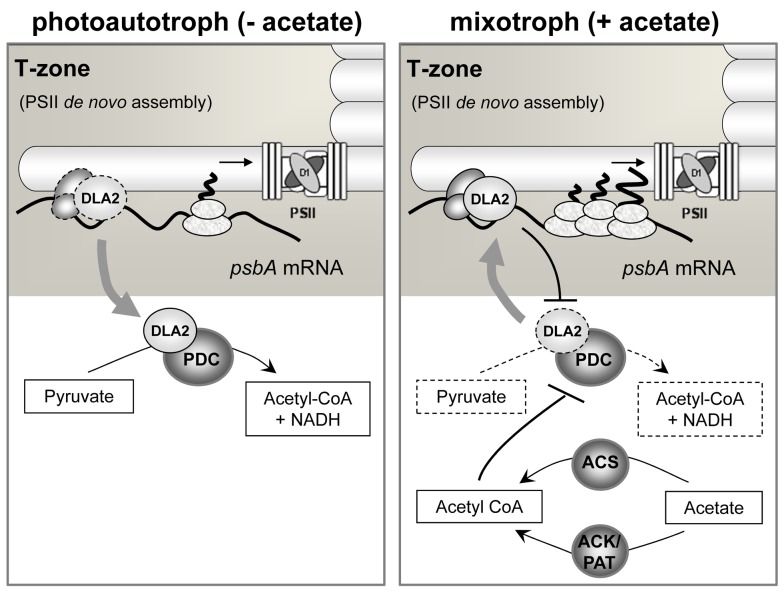
Acetate-dependent regulation of *psbA* gene expression in *C. reinhardtii* by DLA2. Under photoautotrophic conditions (i.e., low acetyl-CoA levels), DLA2 is required as a cpPDC subunit to produce acetyl-CoA (left panel). In contrast, under mixotrophic growth conditions (right panel), acetate is converted into acetyl-CoA by ACS and/or by the ACK/PAT system of *C. reinhardtii* [10]. Accumulating acetyl-CoA levels signal efficient substrate availability for fatty acid synthesis, which causes a product inhibition of the cpPDC and, putatively, its partial disassembly. This leads to an enhanced binding of the DLA2 subunit to the *psbA* mRNA and thus its localization to the T-zone favoring D1 synthesis for de novo assembly of PSII. Reciprocally, the cpPDC activity is decreased by *psbA* mRNA binding to DLA2. For further explanation, see text.

After mass spectrometrical identification of DLA2, we confirmed its in silico predicted chloroplast localization (Figure 3). Results of enzyme activity assays of *DLA2* knock-down lines demonstrated DLA2 to be a functional subunit of the cpPDC (Figures 8 and 9). SEC analyses revealed a *psbA* mRNA-specific DLA2 complex of ca. 700 kDa only in cells cultured mixotrophically (i.e., in the presence of exogenous acetate and light) (Figure 4). In addition, a large >2.8 MDa, RNase-insensitive, DLA2 complex is likely to represent the functional cpPDC (Figures 4 and S3). This complex accumulated predominantly in cells cultured in photoautotrophic conditions and only to a lower level in mixotrophically or heterotrophically grown cells. Which of these complexes is formed, therefore, seems to be dependent on whether light or acetate is available as an energy source. Under photoautotrophic conditions, *C. reinhardtii* chloroplasts depend on the production of acetyl-CoA via cpPDC. Therefore, it is likely that most of DLA2 is in complexes of >2.8 MDa. In contrast, under mixotrophic conditions acetate can be converted into acetyl-CoA by acetyl-CoA synthetase (ACS) and/or by the acetate kinase (ACK)/phosphate acetyltransferase (PAT) system [10]. Accumulating acetyl-CoA then signals substrate availability for fatty acid synthesis, which might cause a product-inhibition of the cpPDC and/or acetylation of its subunits [2],[3]. This, we propose, leads to its partial disassembly, thereby stimulating the light-dependent binding of the *psbA* mRNA to DLA2 and, consequently, the formation of the smaller DLA2 RNP complex (Figure 12). How this light-regulation might be exerted on DLA2 remains to be shown.

The alternate function of DLA2 reported here may be conserved in higher plants. Several hints suggest that cpPDC subunits are present in complexes other than fully assembled cpPDC in higher plants. The analysis of HMW complexes from *A. thaliana* chloroplasts by SEC revealed the chloroplast E2 subunit (LTA2, At3g25860) as a component of two chloroplast complexes, one of >5 MDa and another one of 1–2 MDa [52]. The former most likely represents a fully assembled cpPDC, whereas the latter did not cofractionate with the E1 and E3 subunits. Instead, most E2 coeluted with ribosomal proteins and RNA binding proteins. Likewise, the *A. thaliana* chloroplast E2 subunit has been reported to co-immunoprecipitate with CSP41b, a protein involved in chloroplast RNA metabolism [53]–[55]. Further indications for E2 subunits possessing an additional function besides being a cpPDC subunit are derived from *A. thaliana* T-DNA insertion lines. Assuming that all three cpPDC enzyme components are necessary for its functionality, it is surprising that T-DNA insertions in *E2* genes lead to embryo lethality in the homozygous state, whereas the E3 subunit is dispensable, thereby indicating an essential function of E2 distinct from its role in the cpPDC [56]–[58]. Similarly, an extensive PCR-based search for a DLA2 insertion mutant in an indexed library of *C. reinhardtii* failed, suggesting that DLA2 also is essential in green algae (Bohne, Grossman, and Nickelsen, unpublished results).

### DLA2 Is Involved in the Localization of D1 Synthesis

Consistent with a functional role of the DLA2–RNP complex, the *DLA2*–RNAi lines exhibited growth retardation and reduced D1 synthesis rates under mixotrophic conditions (Figures 6 and 7). Based on results reported here and previously, we propose a role of DLA2 in localizing the *psbA* mRNA to the T-zone for its translation and the synthesis of D1 in de novo PSII biogenesis. First, the existence of mRNA localization factors in the T-zone was suggested by a previous demonstration that the *psbA* mRNA localizes there in the absence of the nascent D1 polypeptide and, hence, any localization information in the polypeptide sequence [16]. Second, we show here that DLA2 is associated with membranes that are distinct from thylakoid membranes in density and within the density range of CTM, a biogenic membrane that is probably localized in the T-zone (Figure 8A, left image, [45]). DLA2 is also associated with stromal thylakoid membranes, another proposed location of *psbA* translation [17]. Third, DLA2 functions in the localization of the *psbA* mRNA in the T-zone (Figure 8C). Together, these results support a model in which the RNA-binding form of DLA2 tethers the *psbA* mRNA to CTM in the T-Zone for translation and cotranslational membrane insertion of D1 for its incorporation into assembling PSII complexes (Figure 12).

While the phenotypes of the *DLA2*–RNAi lines suggest a role in *psbA* translation under mixotrophic conditions, the effects of DLA2 silencing under photoautotrophic conditions appear more pleiotropic. Although PSII levels increase as compared to the wild-type (Figure 7), growth rates remained unaltered despite the requirements for efficient photosynthesis under these conditions (Figure 6). This might be due to counteracting growth limitations caused by reduced cpPDC activity in the absence of acetate. Moreover, this raises the question: What is the molecular basis of the observed differences in the rates of PSII subunit synthesis in the *DLA2*–RNAi lines versus the wild-type in photoautotrophic and heterotrophic conditions (i.e., in the absence of DLA2–RNP complex formation)? Evaluation of the protein pulse-labeling data in Figure 7B revealed that D2 synthesis also increased and decreased concomitantly with D1 synthesis in *DLA2*–RNAi lines under photoautotrophic and heterotrophic growth, again suggesting more pleiotropic effects (Figure S8). In contrast under mixotrophic conditions when DLA2–RNP complex formation occurs, only D1 synthesis but not D2 synthesis was affected, confirming a *psbA*-specific function of DLA2 in the presence of light and acetate (Figure S8). The translational regulation of *psbA* mRNA is complex and has been shown to involve many factors in addition to DLA2 [59]. Nevertheless, based on the presented data, DLA2 appears to be specifically required for adjusting chloroplast gene expression when high levels of reduced carbon energy sources are available to the cell (Figure 12).

### Regulation of cpPDC Activity Via *psbA* mRNA

Little is known about the regulation of cpPDC activity in green algae. DLA2 is constitutively expressed in *C. reinhardtii* under different light conditions, and thus, it is likely that its enzymatic activity is regulated at the posttranslational level (Figure S7). One mechanism to regulate cpPDC activity has been attributed to product inhibition by NADH and acetyl-CoA [11]. Additionally, cpPDC activity is stimulated in the light by an increase of stromal pH as well as Mg^2+^ concentration due to enhanced photosynthetic electron flow (Figure 10A, reviewed in [11]). However, the prediction of a putative RNA binding site overlapping the E3 attachment site within the DLA2 primary amino acid sequence raised the possibility of competitive binding of either E3 or the *psbA* mRNA to this region and, therefore, a further level of cpPDC activity regulation. This idea is supported by the observation that cpPDC activity in the *psbA* deletion mutant *FuD7* is higher than in the PSII mutant *nac2–26* (Figure 10). This is likely due to the lacking inhibitory effect of the *psbA* mRNA in *FuD7*, which can be recovered by the addition of exogenous *psbA* 5′ UTR RNA (Figure 10A,B). This inhibitory effect of *psbA* RNA on cpPDC activity in *FuD7* might suggest a continuous assembly and disassembly of the PDH complex, as it is difficult to imagine how otherwise *psbA* should be able to access the DLA2 subunit, which was reported to be located in the inner core of the enzymatic complex [60]. Disassembled DLA2 could bind RNA and might then be inhibited from re-association with other PDC subunits. Alternatively, the structure of the cpPDC might be different from those known from mitochondrial or bacterial PDCs. This is supported by sedimentation analyses that show that cpPDC forms less defined complexes as compared to mitochondrial or bacterial PDCs [61]. It was speculated that this is due to dissociation of one or more of the component enzymes or association with other enzymes [61].

Taken together, the data indicate a reciprocal light and acetate-dependent regulation of D1 and cpPDC activity, which might guarantee a coordination of protein and lipid synthesis in the course of thylakoid membrane biogenesis. A linkage between carbon and protein metabolism is additionally supported by earlier studies that revealed that chloroplast protein synthesis, including that of D1, can be induced in cells in the dark phase of a diurnal regime after addition of acetate to the medium [62].

### RNA-Binding Activity Might Be an Ancient Feature of dihydrolipoamide acetyltransferases

Our data demonstrate that recombinant E2 proteins from *C. reinhardtii*, human, yeast, and a cyanobacterium all have an intrinsic unspecific RNA binding ability in vitro (Figures 5 and 11). This might point to a very ancient feature of dihydrolipoamide acetyltransferases as RBPs, which is likely to have occurred even before the separation of mitochondrial and chloroplastic homologues. It should be noted, however, that the binding of the human E2 subunit to the provided plant-specific mRNA was very weak as compared to the other tested E2 subunits, and the significance of its RNA binding capacity requires further investigations.

Further indications that RNA binding by E2 subunits is conserved are provided by the human mitochondrial E2 subunit of the mtPDC (Hs-E2). Only recently Hs-E2 has been found to interact with the transcription factor STAT5 (Signal Transducer and Activator of Transcription 5), which was demonstrated to bind mitochondrial DNA [63]. Furthermore, Hs-E2 and STAT5 are translocated to the nucleus under certain conditions, where Hs-E2 is thought to function as a co-activator in STAT5-dependent nuclear gene expression [64]. This raises the possibility that regulation of gene expression by E2 subunits occurs in diverse phylogenetic contexts.

How the RNA binding ability might have been acquired is uncertain, but one might speculate that this occurred before the complex composition of PDC evolved. The ancient E2 enzyme might have had the capacity to bind NAD^+^, a role that is now performed by the E3 subunit of PDC to reoxidize E2-bound dihydrolipoamide produced during catalysis. This is indicated by prediction of a typical NAD^+^/FAD^+^ binding site known as the Rossmann fold within the E3 binding region of many E2 subunits including *C. reinhardtii* DLA2 (Figure S10, [65]). Interestingly, the Rossmann fold, which is typically described as a functional NAD^+^ binding domain, is highly conserved among dehydrogenases and has been reported to be involved in binding of RNA molecules by metabolic enzymes [5],[66]. One well-studied example is the human glyceraldehyde-3-phosphate dehydrogenase (GAPDH), which is described to specifically bind to AU-rich elements within the 3′ UTRs of several mRNAs via its Rossmann fold domain [67],[68]. A wide variety of physiological functions has been attributed to this enzyme–RNA interaction including regulation of mRNA stability, degradation, and/or translation; tRNA and mRNA transport; and as an RNA chaperone (reviewed in [4],[5]). As reported in this study for DLA2, Nagy et al. [67] observed a decreased enzymatic activity of GAPDH upon RNA binding in vitro, which also might indicate a reciprocal regulation between metabolic enzyme activity and RNA-binding (Figure 10B). In conclusion, the available data suggest a complex and coordinated interplay between metabolic pathways and gene expression in the chloroplast.

## Materials and Methods

### Algal Strains and Culture Conditions

As the wild-type *C. reinhardtii* strain, we used CC-406, which has a defective cell wall to allow chloroplast isolation. The chloroplast PSII mutants were *FuD7*, in which the *psbA* genes have been deleted [34], and a *nac2* mutant, which carries *nac2–26* [35] and lacks a stability factor for the *psbD* mRNA. Strains were maintained on 0.8% agar-solidified Tris/acetate/phosphate (TAP) medium [69] at 25°C under constant light (30 µE/m^2^/s) if not indicated otherwise. Liquid cultures were grown under agitation at 25°C to a density of ∼2×10^6^ cells/mL in TAP medium containing 1% sorbitol (TAPS medium) for mixotrophic and heterotrophic growth. Photoautotrophic growth was in high-salt minimal (HSM) medium [69]. Light conditions were as indicated: moderate light (30 µE/m^−2^/s^−1^), high light (200 µE/m^2^/s), or darkness. For GFP import studies and the generation of RNAi lines, the UVM4 expression strain described by Neupert et al. [70] was used.

### Purification of DLA2 and Peptide Sequencing by Mass Spectrometry

Chloroplasts from cell wall–deficient strains carrying the *cw15* mutation were isolated from a discontinuous Percoll gradient (45% to 75%) as described previously [71]. To remove stromal proteins, isolated chloroplasts were osmotically lysed in hypotonic buffer (10 mM tricine/KOH, pH 7.8, 10 mM EDTA, and 5 mM 2-mercaptoethanol) by repeated pipetting. Membrane material was pelleted by ultracentrifugation for 30 min at 100,000 g through a 1 M sucrose cushion in hypotonic buffer in a SW40 rotor (Beckman). Pellets were solubilized in equal volumes of 1.8 M sucrose in hypotonic buffer and floated using an upper 1.3 M sucrose cushion and an additional hypotonic layer (180 min, 4°C, 100,000 g). Thylakoid membranes were taken from the interphase, resuspended in twice the volume of hypotonic buffer, and pelleted again (30 min, 4°C, 100,000 g). The pellet was then lysed in Brij buffer (20 mM tricine/KOH, pH 7.8, 0.12 mM KCl, 0.4 mM EDTA, 10 mM β-mercaptoethanol, 1% Brij-35). After centrifugation (60 min, 4°C, 100,000 g) an aliquot of the resulting supernatant containing solubilized proteins was then applied to a 5 mL heparin–Sepharose 4B (GE Healthcare) column equilibrated with Buffer I (50 mM KCl, 10 mM tricine/KOH, pH 7.8, 10 mM EDTA, and 5 mM 2-mercaptoethanol). Bound proteins were eluted using a discontinuous salt gradient (150 mM, 500 mM, and 1 M KCl in buffer I) after washing the sample with 4 CV of buffer I. Proteins eluting at 150 mM KCl were desalted using Amicon Ultra centrifugal filtration devices (Millipore) with a 10 kDa molecular mass cutoff according to the manufacturer's instructions. The protein solution (in Buffer I) was then applied to a 2 mL poly(A)–Sepharose 4B (GE Healthcare) column equilibrated with buffer I. The column was washed with 4 volumes of buffer I, and bound proteins were eluted with a discontinuous salt gradient (150 mM, 500 mM, and 1 M KCl in buffer I). Prior to use in UV cross-linking assays, all protein fractions were dialyzed against RNA binding buffer (30 mM Tris-HCl, pH 7.0, 50 mM KCl, 5 mM MgCl_2_, and 5 mM 2-mercaptoethanol). Protein concentrations were determined using the Bradford assay (Bio-Rad). For mass spectrometric peptide sequencing, RBP63-containing gel pieces were treated with trypsin (Promega), and the resulting peptides were analyzed on a Q-TOF2 mass spectrometer (Micromass) as described [72].

### Antibody Generation

A fusion protein containing glutathione-S-transferase (GST) and the C-terminal region of the DLA2 protein was used as an antigen for production of a polyclonal rabbit antiserum. For generation of the plasmid expression vector, a DNA fragment encoding the amino acids 391–488 of DLA2 was amplified from genomic DNA by PCR with the primers FWD63*Bam*HI (5′-GGATCCGACCTGGTCAAGCGCGCTCG-3′) and REV63*Sal*I (5′-GTCGACGTTCTCAATCACAGCCTTGA-3′). Attached restriction sites are underlined. The fragment was inserted into the expression vector pGEX4T1 (GE Healthcare) via the *BamH*I and *Sal*I restriction sites. Overexpression and purification of the DLA2–GST fusion protein in the *E. coli* strain BL21 were performed according to the manufacturer's protocol using glutathione–Sepharose 4B (GE Healthcare). A polyclonal antiserum was produced by immunizing rabbits with this protein fraction (Biogenes).

### Chloroplast Preparation and SEC

For analysis of HMW complexes, chloroplasts isolated from *cw15* strains according to Zerges and Rochaix [71] were lysed in nonreducing hypotonic solution (10 mM EDTA, 10 mM tricine-KOH, pH 7.8, and Roche Complete Mini protease inhibitors). Crude thylakoid membranes were separated from soluble proteins by centrifugation on a 1 M sucrose cushion (100,000× g, 30 min, 4°C). The supernatant of this centrifugation step was defined as stromal proteins. Pellets were solubilized in equal volumes of lysis buffer (120 mM KCl, 0.4 mM EDTA, 0.1% Triton X-100, 20 mM tricine, pH 7.8), and insoluble particles were removed by an additional sucrose cushion step. If RNase treatment was required, samples were incubated with 400 U RNase One (Promega)/mg protein for 60 min at 4°C before application to the gel filtration column. Gel filtration samples were loaded through an online filter onto a Superose 6 10/300 GL column (GE Healthcare), and elution was performed at 4°C with buffer containing 50 mM KCl, 2.5 mM EDTA, 5 mM ε-aminocaproic acid, 0.1% Triton X-100, and 20 mM tricine-KOH, pH 7.8, at a flow of 0.3 mL/min using an ÄKTApurifier 10 system (GE Healthcare). Aliquots of each elution fraction were subjected to immunoblotting.

### Preparation of Protein Fractions and Immunoblot Analysis

For protein isolation, cells were placed into 20 mL of liquid TAPS medium and grown under indicated light conditions on a rotary shaker (125 rpm) to mid-log phase (∼5×10^6^–1×10^7^ cells/mL). Cells were harvested by centrifugation and lysed under pipetting in a buffer containing 20 mM KCl, 20 mM tricine, pH 7.8, 0.4 mM EDTA, 5 mM β-mercaptoethanol, and 1% Triton X-100. For cell fractionation, chloroplasts were isolated as described above. Mitochondria were basically prepared according to [73]. Immunoblot analysis was performed using standard procedures. The procedures for subcellular fractionation experiments shown in Figure 8A were described previously [45]. Protein concentrations were determined using the BCA (Pierce) or the Bradford (C. Roth) assay following the manufacturer's instructions.

### GFP Import Studies

A codon-adapted CrGFP has been integrated as *Nde*I/*EcoR*I into the *PsaD* expression vector [74],[75]. This expression cassette was then inserted into the pBC1 vector as *Xho*I/*Xba*I, which contains the *APHVIII* resistance gene under control of the constitutively active *HSP70/RBCS2* promoter regions [76],[77] to result in pBC1-CrGFP. For DLA2–GFP import studies, the coding sequence of the N-terminal amino acids 1–114 including the predicted transit peptide and the complete lipoyl attachment site was PCR-amplified from genomic DNA with the primer pair RBP63 fw (5′-AACATATGCAGGCCACGACCCG-3′)/RBP63 rv (5′-AACATATGCTCGTTGGCGTTTTCGGCCAC-3′), introducing 5′ and 3′ *Nde*I sites (restriction sites underlined). The *Nde*I fragment was then inserted into pBC1–CrGFP to result in pBC1–TP–DLA2–CrGFP. This construct was transformed into UVM4, and positive transformants were selected on TAP plates supplemented with 10 µg/mL paromomycin. As a control for cytosolic CrGFP expression, the pBC1–CrGFP vector was directly transformed into UVM4. GFP fluorescence of transformed cells was observed with a confocal laser scanning system (Zeiss LSM 51 Meta).

### In Vitro Synthesis of RNA and RNA Binding Studies to Recombinant DLAs

For expression of recombinant DLA2 protein from *C. reinhardtii*, a 1,389 bp fragment encoding the C-terminal amino acids 32–494 was PCR-amplified from EST clone MXL069g06 (Kazusa DNA Research Institute) using the primer pair RBP63–pQEfw*Bam*HI (5′-aaggatccAACGCGGTCAAGGATG-3′)/RBP63-pQErev*Sal*I (5′- gtcgacTTAGAACAGCAGCTGGTCGG-3′) and inserted into the plasmid pQE30 (Qiagen) via the *BamH*I/*Sal*I restriction sites to yield the plasmid pQE–DLA2. Expression was accomplished in *E. coli* M15 cells (Stratagene). Cells were grown to an OD_600_ of 0.5–0.6, and protein expression induced by addition of IPTG to a final concentration of 1 mM followed by growth at 37°C for 3 h. The recombinant protein was purified according to the GE Healthcare protocol for purification of histidine-tagged recombinant proteins under native conditions using Ni Sepharose 6 Fast Flow (GE Healthcare). Primers used for cloning of other His-E2 fusion proteins excluding predicted signal and transit peptides are as follows: *Synechocystis* sp. 6803 Fw (5′-aaggatccATTTACGACATTTTCATGCC-3′)/Rev (5′-gtcgacGTCAAAGACTGGGCATTC-3′), *Saccharomyces cerevisiae* Fw (5′-ggatccCCAGAGCACACCATTATTG-3′)/Rev (5′-gtcgacTCACAATAGCATTTCCAAAGG-3′), and *Homo sapiens* Fw (5′-ggatccCCGCATCAGAAGGTTCCATTG-3′)/Rev (5′-gtcgacAGTGTGACCTGGGAGAGTTTA-3′). For cloning of the expression vector for cyanobacterial PratA (*slr2048*), the CDS (aa 38–383) was amplified using the following primers: Ss_PratA_fw01 (5′-ctaggatccAATCTTCCTGACGTTACCC-3′)/Ss_PratA_rv01 (5′-ctactgcagTTAGAGATTATCCAGCTTTTCTTGG-3′) and cloned via *BamH*I/*Pst*I sites into the vector pET28b SUMO-Ser. The full length CDS of RBP40 was amplified with primers BamHI–RBP40 (5′-aaggatccATGCTGACCTTGAGACGTGC-3′)/RB38DN44revSalI (5′-ttgtcgacCTAGTAGCGGGCGCCC-3′) and cloned into the vector pQE30 via *BamH*I/*Sal*I sites.

Expression and purification were as described above with some minor changes: Expression for Hs-E2 was performed overnight at 17°C (0.5 mM IPTG), Syn-E2 for 5 h at 25°C, Sc-E2 for 5 h at 18°C, and RBP40 for 5 h at 30°C. PratA was expressed in BL21 DE3 cells overnight at 12°C. Concentrations of recombinant proteins were determined along with a BSA dilution series.

In vitro synthesis of RNA and UV cross-linking experiments were basically performed as described by Zerges and Rochaix [71]. DNA templates for the in vitro synthesis of *rbcL*, *psbD*, and *psbA* leader RNA probes were generated by PCR using the following primers: T7rbcL5 (5′-gtaatacgactcactatagggTATGCTCGACTGATAAGAC-3′)/rbcL3 (5′-CTGCTTTAGTTTCTGTTTGTGGAACC-3′); T7psbD5 (5′-gtaatacgactcactatagggCCACAATGATTAAAATTAAA-3′)/psbDUTR3 (5′-ACCGATCGCAATTGTCAT-3′); and T7psbA5 (5′-gtaatacgactcactatagggTACCATGCTTTTAATAGAAG-3′)/2054-psbA (5′-GATCCATGG TCATATGTTAATTTTTTTAAAG-3′). Each template contained the promoter of the T7 RNA polymerase (written in lowercase letters in the fw-primer sequence) fused to the 5′ end of the described fragments. A total of 0.5 µg of the PCR products were transcribed in vitro by T7 RNA Polymerase (Fermentas) in a 20 µl reaction in the presence of 20 U RNase inhibitor (Fermentas), 40 µCi of α-^32^P-UTP (3,000 Ci/mmol; Hartmann Analytic), 30 µM nonradiolabeled UTP, and 0.5 mM each of ATP, CTP, and GTP according to the manufacturer's protocol. We added 1 U of RNase-free DNase (Promega), and the reaction was incubated for an additional 15 min at 37°C. Unincorporated nucleotides were removed using a MicroSpin S-200 HR column (GE Healthcare). The reactions were extracted once with phenol-chloroform and ethanol precipitated in the presence of ammonium. Binding reactions (20 µl) were performed at RT for 5 min and contained 20 mM HEPES/KOH, pH 7.8, 5 mM MgCl_2_, 60 mM KCl, and 200 ng or 10 ng protein. Each reaction contained 50–100 kcpm of ^32^P-RNA probe. For competition experiments, protein and RNA probe were used in equimolar amounts or in 5-fold to 200-fold excess of cold RNA. Radiolabeled RNA and nonlabeled competitors were mixed prior to the addition of proteins in competition experiments. Quantification of competitor RNAs was performed by measuring the incorporation of low levels of radioactivity into transcripts. Subsequent exposure to a 254 nm UV irradiation of 1 J/cm_2_ using a Stratalinker UV cross-linker (Stratagene) covalently cross-linked the RNA probe and bound proteins. After irradiation, the nonbound ^32^P-RNA probes were digested by treatment with 10 U RNase One (Promega) for 20 min at 37°C. Samples were fractionated by SDS-PAGE and analyzed by autoradiography or phosphorimaging.

The *K_D_* was determined as described by Ostersetzer et al. [38]. Increasing amounts of recombinant DLA2 protein were incubated for 15 min at RT with in vitro transcribed ^32^P-labeled *psbA* mRNA (6.7 pM) in 30 µl reactions in the same binding buffer used for UV cross-linking assays. Subsequently, the reactions were filtered through stacked nitrocellulose (Reprobe nitrocellulose plus, 0.45 µm; Applichem) and nylon membranes (Nylon plus, 0.45 µm; C. Roth) using a dot blot apparatus (Minifold SRC96, Schleicher & Schuell). The membranes were washed once with 100 µl of binding buffer, dried, and subjected to phosphorimaging and quantitation with AlphaEase software (Alpha Innotech Corporation).

### Generation of RNAi Lines

To create DLA2-deficient mutants of *C. reinhardtii*, we used the RNAi system previously described by Rohr et al. [41]. For the generation of an inverted repeat construct specific for *DLA2* RNA, a 400 bp fragment corresponding to the last exon and part of the 3′ UTR of *DLA2* was amplified by PCR using genomic DNA as a template with the primers 5/63 SHE 5′-GTCGACAAGCTTGAATTCCAACTGGGCTCA-3′ and 3/63 400 B 5′-GGATCCGCTAACCCTGCAGCCCACCT-3′, which add *Sal*I, *Hind*III, *Eco*RI, or *Bam*HI restriction sites, respectively (restriction sites underlined). A longer 600 bp fragment containing an additional 200 bp of the 3′ UTR that functioned as a spacer for the inverted repeat was amplified using the primers 5/63 SHE and 3/63 600 B (*BamH*I) 5′-GGATCCGGCATTCAAGCCACCCTGCT-3′. These two fragments were ligated and cloned as an inverted repeat (with the central spacer) into the *EcoR*I site of the vector NE537 [41]. The UVM4 strain was transformed with the resulting construct, kept for 2 d in liquid culture (TAP +1.5 mM L-tryptophan) in dim light, and then plated on TAP plates containing 5 µg/mL paromomycin and 1.5 mM L-tryptophan [70]. *C. reinhardtii* cells transformed with the empty NE537 vector served as a control (WT-NE). At intervals of 2 wk, colonies were transferred to TAP plates containing 5 and then 10 µM 5-fluoroindole (5-FI). Plates were kept in low light (∼10 µE/m^2^/s^−1^) under a yellow foil (Q-MAX 010 medium yellow, Multi-Lite, Hamburg, Germany), which filters out wavelengths of light between 400 and 470 nm to inhibit photodegradation of tryptophan and 5-FI.

### FISH and IF Staining

FISH and IF were performed according to Uniacke et al. [78]. The *psbA* FISH probes were labeled with Alexa Flour 488, and the IF staining involved Alexa Fluor 568 conjugated anti-rabbit secondary antibody (Invitrogen). Images were captured on a Leica DMI6000B microscope (Leica Microsystems) using a 40×/0.75 objective, a Hamamatsu OrcaR2 camera, and Volocity acquisition software (Perkin Elmer). For each condition, ≥20 cells were observed.

### ^35^S Pulse Labeling of Proteins

Chlamydomonas liquid cultures were grown in TAPS or HSM medium to a density of ∼1–2×10^6^ cells/mL, pelleted by centrifugation (10 min, 4°C, 1,000× g), resuspended in the same medium in which all sulfur-containing ingredients were replaced by the respective chloride salts (TAPS-S/HSM-S), and incubated for 16 h at 23°C in the light. Cells were pelleted, washed, and resuspended in TAPS-S/-T or HSM-S/-T (lacking both sulfur salts and trace elements), respectively, and grown under indicated light conditions for 2 h. Cells were then washed again and resuspended in TAPS-S/-T or HSM-S/-T to a concentration of 80 µg chlorophyll per mL. Aliquots (225 µl) of the cell suspension were incubated with cycloheximide (10 µg/mL) for 10 min. Subsequently, 100 µCi H_2_^35^SO_4_ (Hartmann Analytic) was added to each, followed by incubation for 15 min in the light as before. After centrifugation, sedimented cells were frozen in liquid nitrogen. Cells were resuspended in 10 mM HEPES-KOH, pH 7.5, 10 mM EDTA in the presence of CompleteMini protease inhibitors (Roche) and disrupted by sonication (30 s, RT). The homogenate was then centrifuged at 20,000 g for 30 min. The pellet was resuspended in 10 mM HEPES-KOH, pH 7.5, 10 mM EDTA. Samples were fractionated by electrophoresis on 16% SDS-polyacrylamide gels containing 8 M urea. Radioactive protein signals were detected on the dried gel by phosphorimaging. Significance of difference between the mean D1/AtpA/B signal ratio for each RNAi line and WT-NE (100%) was determined with a one-sample *t* test (*p*<0.05).

### Assay of cpPDC Activity

Cells were lysed in a buffer containing 25 mM MgCl_2_, 100 mM tricine (pH 8.0), and 0.1% Triton X-100 at 4°C by sonication. Insoluble material was removed by centrifugation (10 min, 4°C, 10,000 g). cpPDC activity was measured photometrically at 23°C using a Pharmacia Biotech Ultrospec 3000 spectrophotometer. The assay was based on that described by Qi et al. [79] and performed under conditions that favor the activity of the cpPDC over mtPDC (high Mg^2+^ concentration, alkaline pH; [61],[80]).

The reaction mixture used contained 0.1 mM TPP, 5 mM MgCl_2_, 2 mM NAD^+^, 0.1 mM CoA, 3 mM cysteine, 0.05% Triton X-100, 0.1 M tricine (pH 8.0), and 200 µg of proteins in a final volume of 0.990 mL. Reactions were initiated by the addition of 1 µmol sodium pyruvate in a volume of 10 µl, and the change in absorbance at 340 nm caused by NADH production was followed for 2 min. If the influence of DCMU was tested, it was added to a final concentration of 20 µM 3 h prior to cell harvest. An equivalent volume of ethanol, the solvent for the DCMU stock, was used instead of DCMU for control strains. For pre-incubation of cell lysates with *psbA* or *rbcL* mRNAs, 5′ UTRs were in vitro transcribed as described above. Lysates were prepared as described above in the presence of 250 U/mL RNase inhibitor (Fermentas). Protein samples (200 µg) were incubated with 150 or 450 pmol RNA in a final volume of 130 µl for 10 min at RT prior to the PDC activity assay. Unpaired two-sample *t* tests were used to determine whether the mean activity under each condition is significantly different from that in the absence of RNA (*p*<0.05).

If cpPDC activity tests were performed with size exclusion fractions, SEC elution was performed in 60 mM KCl, 5 mM MgCl_2_, and 100 mM tricine-KOH, pH 7.8, and 0.05%. We used 300 µl of 1.2 ml fractions for the assay as described above.

## Supporting Information

Figure S1 Determination of the copy number of the *DLA2* gene in *C. reinhardtii* by genomic Southern blotting. (A) Schematic representation of the genomic region containing the *DLA2* gene (shown: chromosome 3, 1415567–1424567 in JGI v4). Enzyme restriction sites leading to respective restriction fragments shown under (B) are indicated. Grey bar represents the position of the DNA probe used for Southern hybridization. (B) Southern blot. A total of 10 µg of total *C. reinhardtii* DNA from wild-type strain *cw15* were digested with the restriction enzymes *Nco*I, *Nhe*I, and *Pst*I; separated on 0.8% agarose gels; transferred to a nylon membrane; and hybridized with a gene-specific probe indicated under (A). The digoxigenin-11-dUTP (Roche Diagnostics)–labeled DNA-probe hybridizing to the 5′ region of the *DLA2* gene was generated by PCR using the primer pair Fw (5′-*aacat*atgCAGGCCACGACCCG-3′)/Rev (5′-*aacatatg*CTCGTTGGCGTTTTCGGCCAC-3′). The hybridization signals were visualized by chemiluminescence detection using anti-DIG antibody conjugated with alkaline phosphatase and the substrate, CDP-STAR (Roche Diagnostics). Sizes of respective signals are indicated on the right.
(TIFF)

Figure S2 Size exclusion analysis of stromal DLA2 complexes. Stromal proteins (treated with RNase or not) from wild-type CC-406 grown under indicated conditions were separated by SEC (A–C). Fractions 1 to 14 were subjected to protein gel blot analysis using the DLA2 antibody. Molecular masses were calculated by parallel analysis of high molecular mass calibration markers. A quantitation of DLA2 signal intensities, with error bars calculated from three independent experiments, was performed as described in Figure 4 and is presented below each panel.
(TIF)

Figure S3 cpPDC activity of SEC fractions. PDC activity was measured from SEC-separated crude thylakoids (A) and stroma (B) fractions 1–6 of mixotrophically grown wild-type cells. Prior to SEC, proteins were treated with RNase or not as indicated. The highest cpPDC activity measured in fraction 1 was set to 100%. Mean values and standard deviations of three independent experiments are shown. Corresponding DLA2 protein levels as quantified in Figure 4C are indicated.
(TIFF)

Figure S4 Immunoprecipitation of DLA2 protein and bound RNA. (A) Immunoprecipitation of DLA2 protein. Proteins from crude thylakoids (cTs) of 5 L mixotrophically grown *C. reinhardtii* cell-wall-deficient wild-type cells were solubilized in lysis buffer (10 mM tricine/KOH, pH 7.8, 10 mM EDTA, 1% Triton X-100, and Roche Complete mini protease inhibitor cocktail). For immunoprecipitation with DLA2 antiserum (α-DLA2) or preimmune serum (Pre) coupled to protein A Sepharose (GE Healthcare), lysates were diluted in TBS buffer to reach a Triton X-100 concentration of 0.1%. Proteins were extracted from precipitates (P) and supernatants (S), and the entire bead aliquots were subjected to SDS-PAGE and Western blot analysis using the DLA2 antibody. Molecular weight markers are indicated on the left. Quantification of signals revealed that less than 0.2% of the total DLA2 protein was precipitated (not shown). (B) *psbA* mRNA can be co-immunoprecipitated with DLA2 proteins from cells grown under mixotrophic conditions. Solubilized crude thylakoid proteins of heterotrophically or mixotrophically grown *C. reinhardtii* cell-wall-deficient wild-type cells were used for immunoprecipitation reactions with DLA2 antiserum (α-DLA2) or preimmune serum (Pre) as described in (A). RNAs were extracted from precipitates (P_αDLA2_ and P_Pre_) with TriReagent (Sigma-Aldrich) according to the manufacturer's instructions, and equal proportions were subjected to dot-blot hybridization using dig-labelled DNA probes indicated on the top. Dig-labeled DNA probes comprising sequences of the coding regions of the indicated genes were PCR-amplified from cDNA (*psbA*) or total DNA using DIG-11-dUTP (Roche Diagnostics) and gene-specific primer pairs: psbA, psbA-1 (5′-TCTAGCCTATGGGCTCGT-3′)/psbA-2 (5′-ACCGAAACGGTAACCTTC-3′); atpB, creatpB3int (5′-gattctaatgggtcaaccg-3′)/creatpB5int (5′-ccaacagaaggtttaatgcg-3′); and rbcL, crerbcL5int (5′-CGGTTTAACAAGTCTTGACC-3′)/crerbcL3int (5′-CACCGAACTGAAGACATGC-3′). Visualization of hybridization signals was performed by ECL using AP-conjugated anti-DIG-antibody and CDP* substrate (Roche Diagnostics).
(TIFF)

Figure S5 *psbA* mRNA accumulation in *iDLA2* lines under different growth conditions. For Northern blot analyses (upper panel) of indicated RNAi lines (*iDLA2*-1, -2, -3), and the control strain (WT-NE) total RNA was extracted by using TRI Reagent (Sigma-Aldrich) according to the manufacturer's instructions. A total of 3 µg were separated electrophoretically, blotted onto a nylon membrane, and hybridized with a *psbA* probe. The probe was produced by PCR by using DIG-11-dUTP (Roche Diagnostics) and the primers 277 (5′-GAGCAAAGTTACTGTTTCTATGGA-3′)/278 (5′-TGAGCCATCTTTCATCAACC-3′) on *psbA* cDNA. The hybridization signals were visualized by chemiluminescent detection using anti-DIG antibody conjugated with alkaline phosphatase and the substrate, CDP-STAR (Roche Diagnostics). The ethidium bromide-stained gel is shown as loading control (lower panel). *FuD7*, *psbA* deletion mutant.
(TIFF)

Figure S6 Lipid accumulation in *iDLA2* lines. Lipids from whole cells were isolated according to Rengstl et al. [83] from 50 mL photoautotrophically grown wild-type transformed with the empty vector NE 537 (WT-NE) or *DLA2*-RNAi lines (*iDLA2*-1, -2, -3). After extraction, lipids were separated by thin-layer chromatography visualized by incineration. The mayor chloroplast lipids monogalactosyldiacylglycerol (MGDG) and digalactosyldiacylglycerol (DGDG) are indicated.
(TIFF)

Figure S7 DLA2 protein accumulation under different growth conditions. A total of 30 µg of whole cell proteins from a cell-wall-deficient wild-type strain grown under mixotrophic (M), heterotrophic (H), or photoautotrophic (P) conditions were separated by SDS-PAGE and subjected to immunoblot analysis. The blot was probed with antibodies against DLA2 and the large subunit of Rubisco (RbcL).
(TIF)

Figure S8 Quantification of D1 and D2 protein synthesis rates in *DLA2*–RNAi lines under various growth conditions. D1 and D2 protein synthesis rates were determined as described in Figure 7. Quantification of signals was performed by using AlphaEaseFC software (Alpha Innotech Corp.) and calculating the ratio of D1 or D2, respectively, over AtpA/B. Values obtained for WT-NE were set to 100%. Growth conditions as indicated.
(TIFF)

Figure S9 Prediction of RNA binding residues in E2 subunits of various organisms. Possible RNA binding residues within the amino acid sequence of E2 subunits from *C. reinhardtii* (A), *Synechocystis* sp. PCC 6803 (B), *S. cerevisiae* (C), and *H. sapiens* (D) were predicted by using the RNAbindR software with set “optimal prediction” (http://bindr.gdcb.iastate.edu/RNABindR) [84]. Plus signs below the amino acid sequences indicate the predicted RNA-binding residues, whereas minus signs indicate a low probability of RNA binding. The conserved domains for lipoyl attachment, E3 binding, and the 2-oxo acid dehydrogenase catalytic domain are marked above the sequence by grey, black, and dark grey bars, respectively. Transit and signal peptides as predicted by TargetP [23]. Accession numbers as in Figure 2.
(TIFF)

Figure S10 Alignment of predicted Rossmann folds within the amino acid sequence of PDC-E2 subunits and human GAPDH. For accession numbers, see Figure 2. *H. sapiens*, HsGAPDH (accession no: NP_002037). Highly conserved residues are written in bold. The Rossmann fold consensus sequence is shown below the alignment as given on the NIH MBI laboratory prediction server (http://nihserver.mbi.ucla.edu//cgi-bin/Rossmann/Rossmann.cgi).
(TIFF)

## Abbreviations

5-FI: 5-fluoroindole
ACK: acetate kinase
ACS: acetyl-CoA synthetase
AOX: alternative oxidase
co-IPs: co-immunoprecipitations
cpPDC: chloroplast pyruvate dehydrogenase complex
DLA: dihydrolipoamide acetyltransferase
FISH: fluorescence in situ hybridization, GAPDH, glyceraldehyde-3-phosphate dehydrogenase
GST: glutathione-S-transferase
HSM: high-salt minimal
HMW: high molecular weight
IF: immunofluorescence
mtPDC: mitochondrial pyruvate dehydrogenase complex
PAT: phosphate acetyltransferase
PDH: pyruvate dehydrogenase
PSI: photosystem I
PSII: photosystem II
RBP: RNA binding protein
RNP: ribonucleoprotein
SEC: size exclusion chromatography
STAT5: Signal Transducer and Activator of Transcription 5
TAP: Tris/acetate/phosphate
WT: wild-type
