# Supplementary figures and images for: Reciprocal Regulation of Protein Synthesis and Carbon Metabolism for Thylakoid Membrane Biogenesis
Source: PLoS Biol. 2013 Feb 12;11(2):e1001482. doi: 10.1371/journal.pbio.1001482 (PMC3570535; doi:10.1371/journal.pbio.1001482)

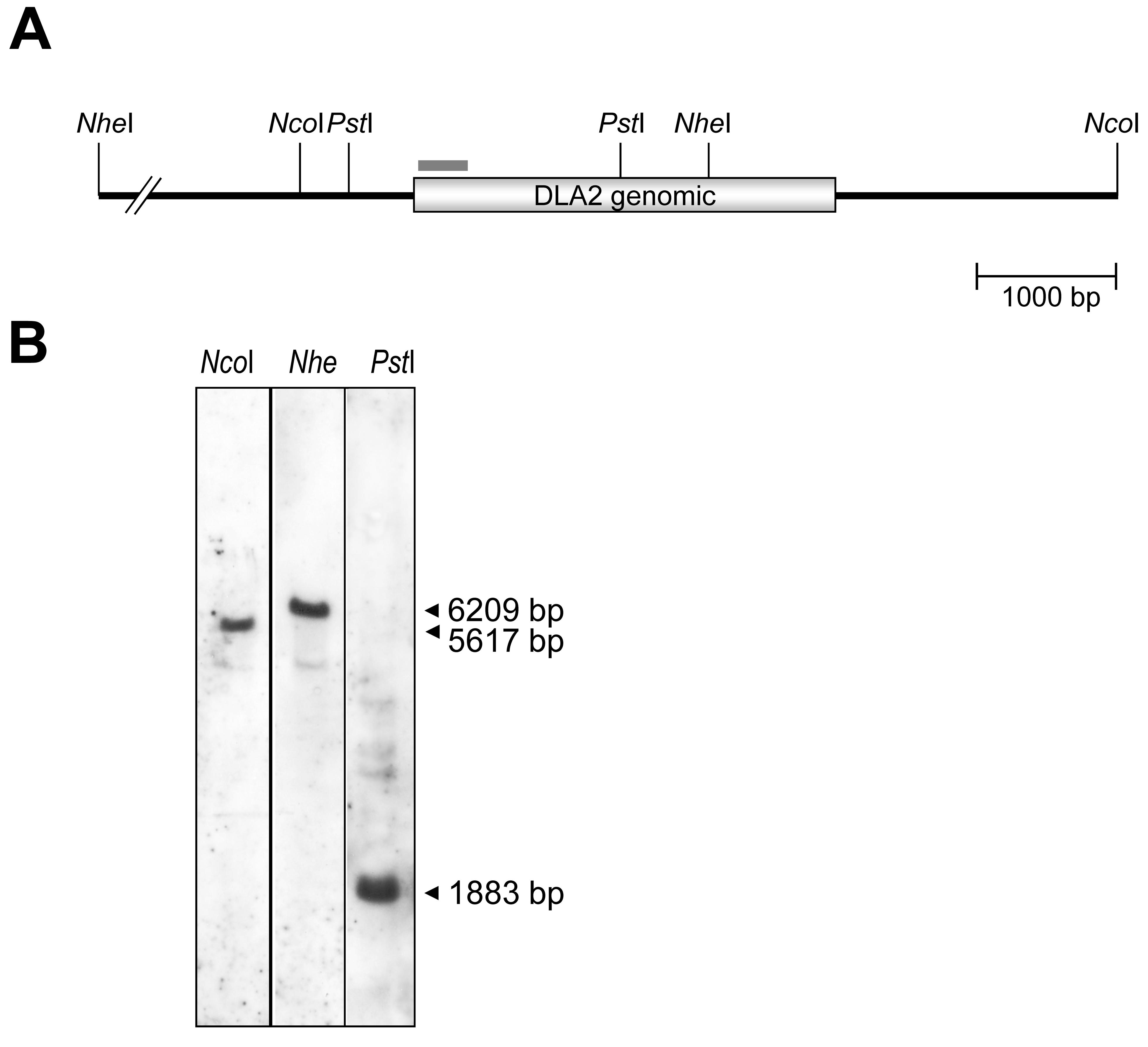

Supplement: Figure S1 — Determination of the copy number of the DLA2 gene in C. reinhardtii by genomic Southern blotting. (A) Schematic representation of the genomic region containing the DLA2 gene (shown: chromosome 3, 1415567–1424567 in JGI v4). Enzyme restriction sites leading to respective restriction fragments shown under (B) are indicated. Grey bar represents the position of the DNA probe used for Southern hybridization. (B) Southern blot. A total of 10 µg of total C. reinhardtii DNA from wild-type strain cw15 were digested with the restriction enzymes NcoI, NheI, and PstI; separated on 0.8% agarose gels; transferred to a nylon membrane; and hybridized with a gene-specific probe indicated under (A). The digoxigenin-11-dUTP (Roche Diagnostics)–labeled DNA-probe hybridizing to the 5′ region of the DLA2 gene was generated by PCR using the primer pair Fw (5′-aacatatgCAGGCCACGACCCG-3′)/Rev (5′-aacatatgCTCGTTGGCGTTTTCGGCCAC-3′). The hybridization signals were visualized by chemiluminescence detection using anti-DIG antibody conjugated with alkaline phosphatase and the substrate, CDP-STAR (Roche Diagnostics). Sizes of respective signals are indicated on the right. (TIFF) [file pbio.1001482.s001.tiff]

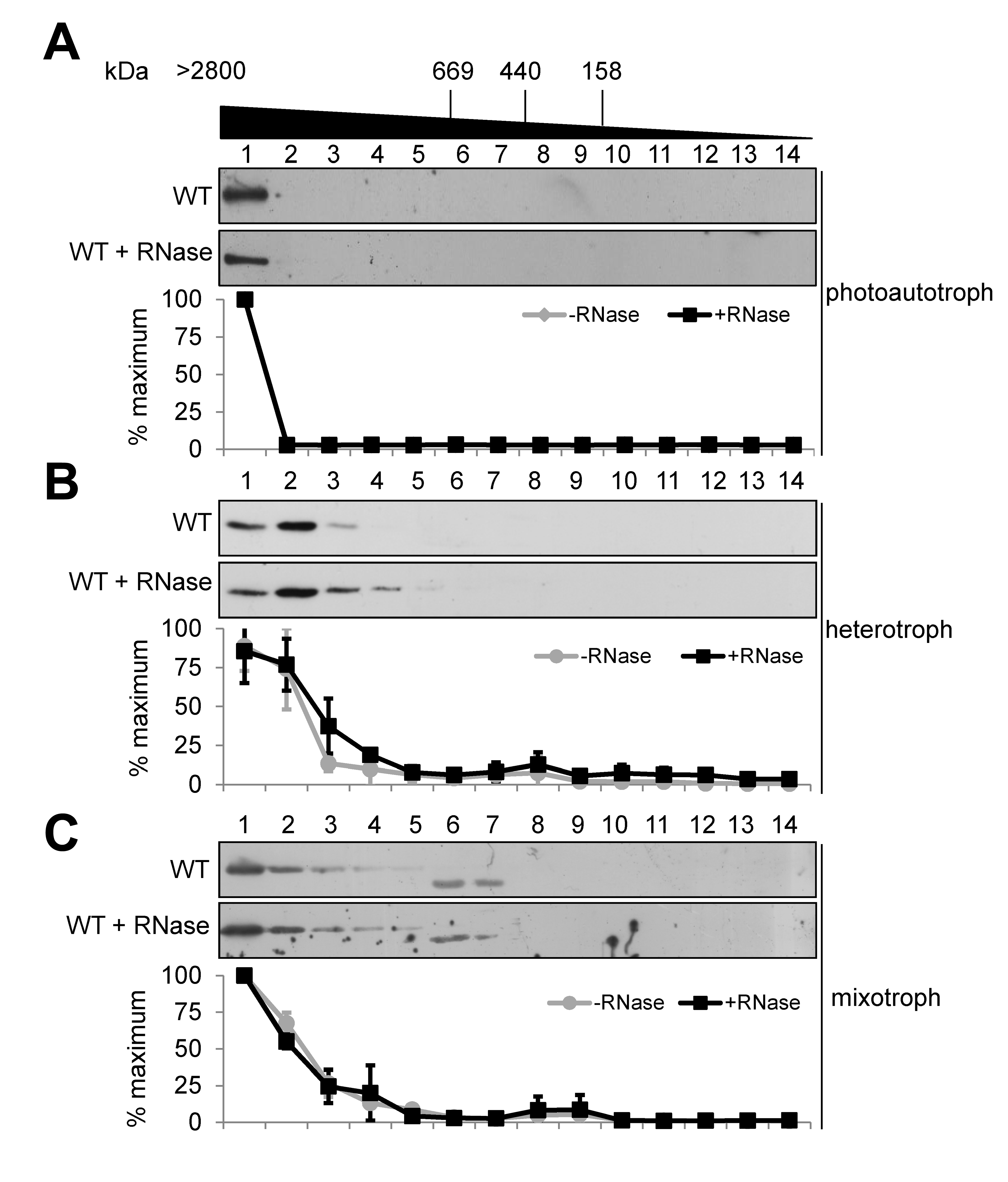

Supplement: Figure S2 — Size exclusion analysis of stromal DLA2 complexes. Stromal proteins (treated with RNase or not) from wild-type CC-406 grown under indicated conditions were separated by SEC (A–C). Fractions 1 to 14 were subjected to protein gel blot analysis using the DLA2 antibody. Molecular masses were calculated by parallel analysis of high molecular mass calibration markers. A quantitation of DLA2 signal intensities, with error bars calculated from three independent experiments, was performed as described in Figure 4 and is presented below each panel. (TIF) [file pbio.1001482.s002.tif]

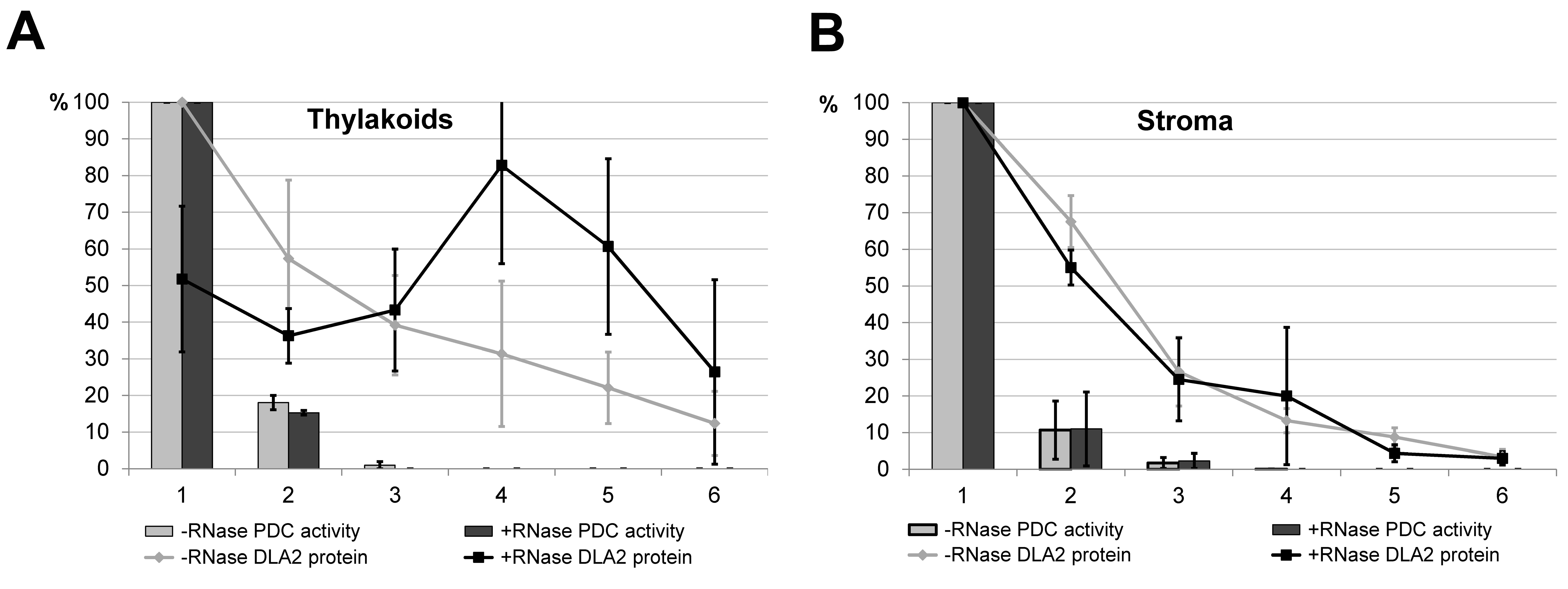

Supplement: Figure S3 — cpPDC activity of SEC fractions. PDC activity was measured from SEC-separated crude thylakoids (A) and stroma (B) fractions 1–6 of mixotrophically grown wild-type cells. Prior to SEC, proteins were treated with RNase or not as indicated. The highest cpPDC activity measured in fraction 1 was set to 100%. Mean values and standard deviations of three independent experiments are shown. Corresponding DLA2 protein levels as quantified in Figure 4C are indicated. (TIFF) [file pbio.1001482.s003.tiff]

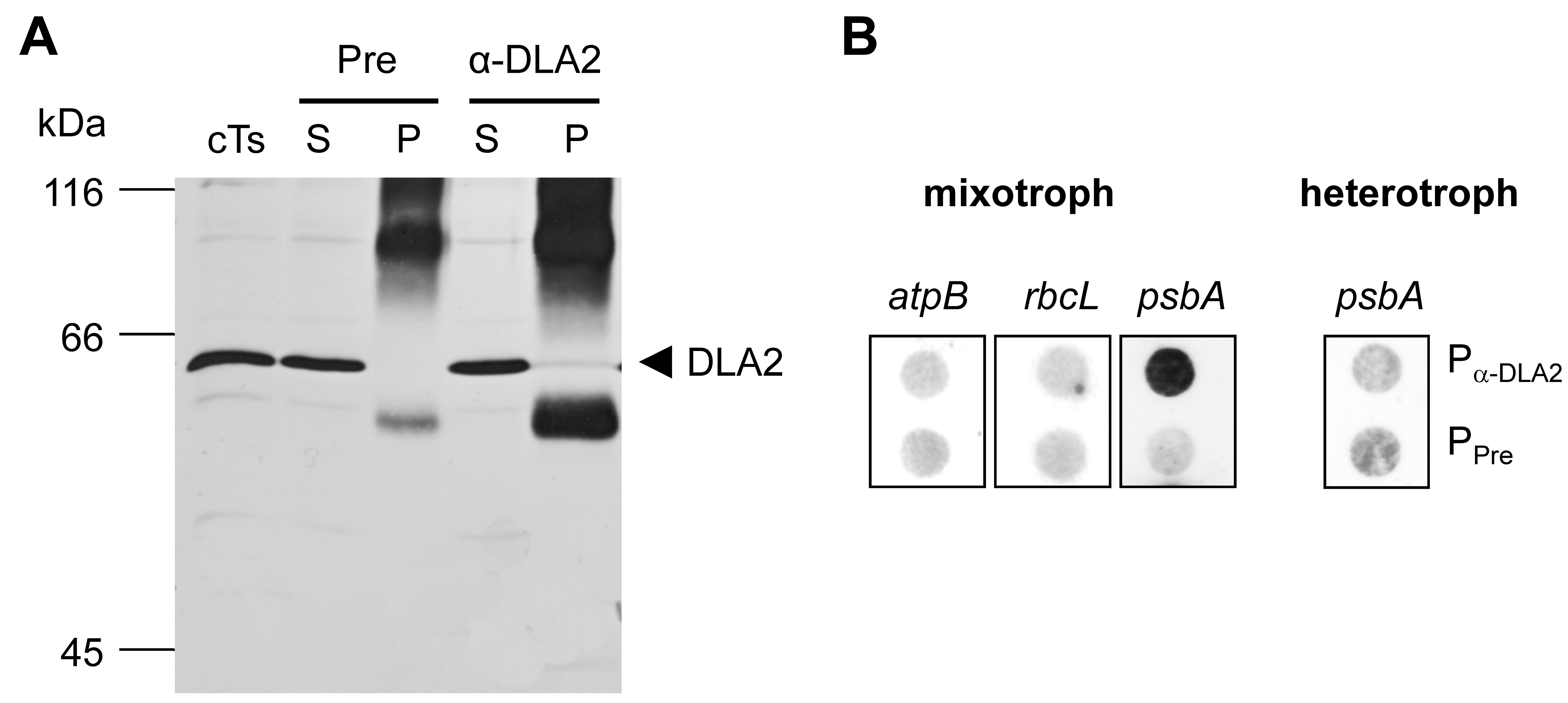

Supplement: Figure S4 — Immunoprecipitation of DLA2 protein and bound RNA. (A) Immunoprecipitation of DLA2 protein. Proteins from crude thylakoids (cTs) of 5 L mixotrophically grown C. reinhardtii cell-wall-deficient wild-type cells were solubilized in lysis buffer (10 mM tricine/KOH, pH 7.8, 10 mM EDTA, 1% Triton X-100, and Roche Complete mini protease inhibitor cocktail). For immunoprecipitation with DLA2 antiserum (α-DLA2) or preimmune serum (Pre) coupled to protein A Sepharose (GE Healthcare), lysates were diluted in TBS buffer to reach a Triton X-100 concentration of 0.1%. Proteins were extracted from precipitates (P) and supernatants (S), and the entire bead aliquots were subjected to SDS-PAGE and Western blot analysis using the DLA2 antibody. Molecular weight markers are indicated on the left. Quantification of signals revealed that less than 0.2% of the total DLA2 protein was precipitated (not shown). (B) psbA mRNA can be co-immunoprecipitated with DLA2 proteins from cells grown under mixotrophic conditions. Solubilized crude thylakoid proteins of heterotrophically or mixotrophically grown C. reinhardtii cell-wall-deficient wild-type cells were used for immunoprecipitation reactions with DLA2 antiserum (α-DLA2) or preimmune serum (Pre) as described in (A). RNAs were extracted from precipitates (PαDLA2 and PPre) with TriReagent (Sigma-Aldrich) according to the manufacturer's instructions, and equal proportions were subjected to dot-blot hybridization using dig-labelled DNA probes indicated on the top. Dig-labeled DNA probes comprising sequences of the coding regions of the indicated genes were PCR-amplified from cDNA (psbA) or total DNA using DIG-11-dUTP (Roche Diagnostics) and gene-specific primer pairs: psbA, psbA-1 (5′-TCTAGCCTATGGGCTCGT-3′)/psbA-2 (5′-ACCGAAACGGTAACCTTC-3′); atpB, creatpB3int (5′-gattctaatgggtcaaccg-3′)/creatpB5int (5′-ccaacagaaggtttaatgcg-3′); and rbcL, crerbcL5int (5′-CGGTTTAACAAGTCTTGACC-3′)/crerbcL3int (5′-CACCGAACTGAAGACATGC-3′). Visualization of hybridizat [file pbio.1001482.s004.tiff]

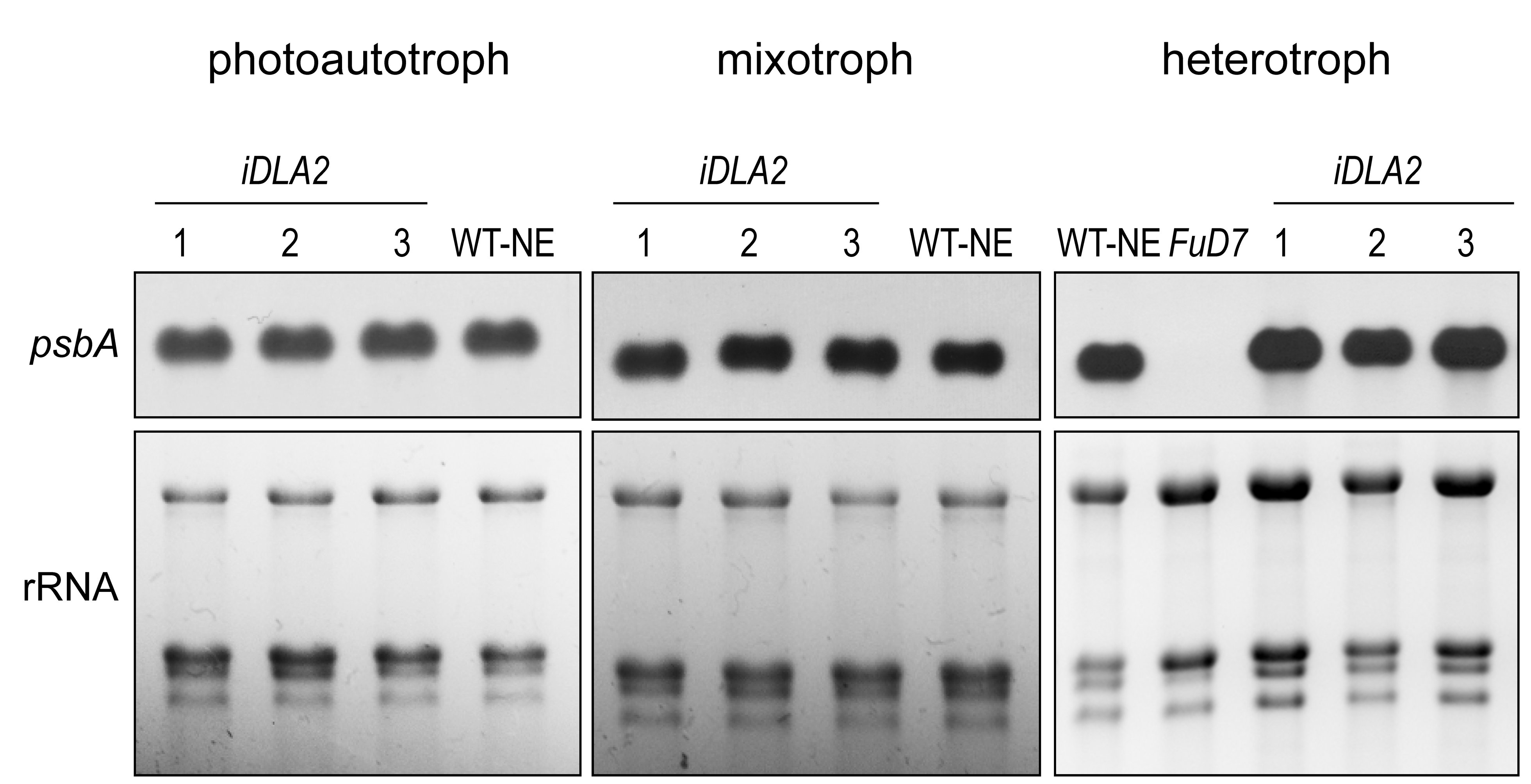

Supplement: Figure S5 — psbA mRNA accumulation in iDLA2 lines under different growth conditions. For Northern blot analyses (upper panel) of indicated RNAi lines (iDLA2-1, -2, -3), and the control strain (WT-NE) total RNA was extracted by using TRI Reagent (Sigma-Aldrich) according to the manufacturer's instructions. A total of 3 µg were separated electrophoretically, blotted onto a nylon membrane, and hybridized with a psbA probe. The probe was produced by PCR by using DIG-11-dUTP (Roche Diagnostics) and the primers 277 (5′-GAGCAAAGTTACTGTTTCTATGGA-3′)/278 (5′-TGAGCCATCTTTCATCAACC-3′) on psbA cDNA. The hybridization signals were visualized by chemiluminescent detection using anti-DIG antibody conjugated with alkaline phosphatase and the substrate, CDP-STAR (Roche Diagnostics). The ethidium bromide-stained gel is shown as loading control (lower panel). FuD7, psbA deletion mutant. (TIFF) [file pbio.1001482.s005.tiff]

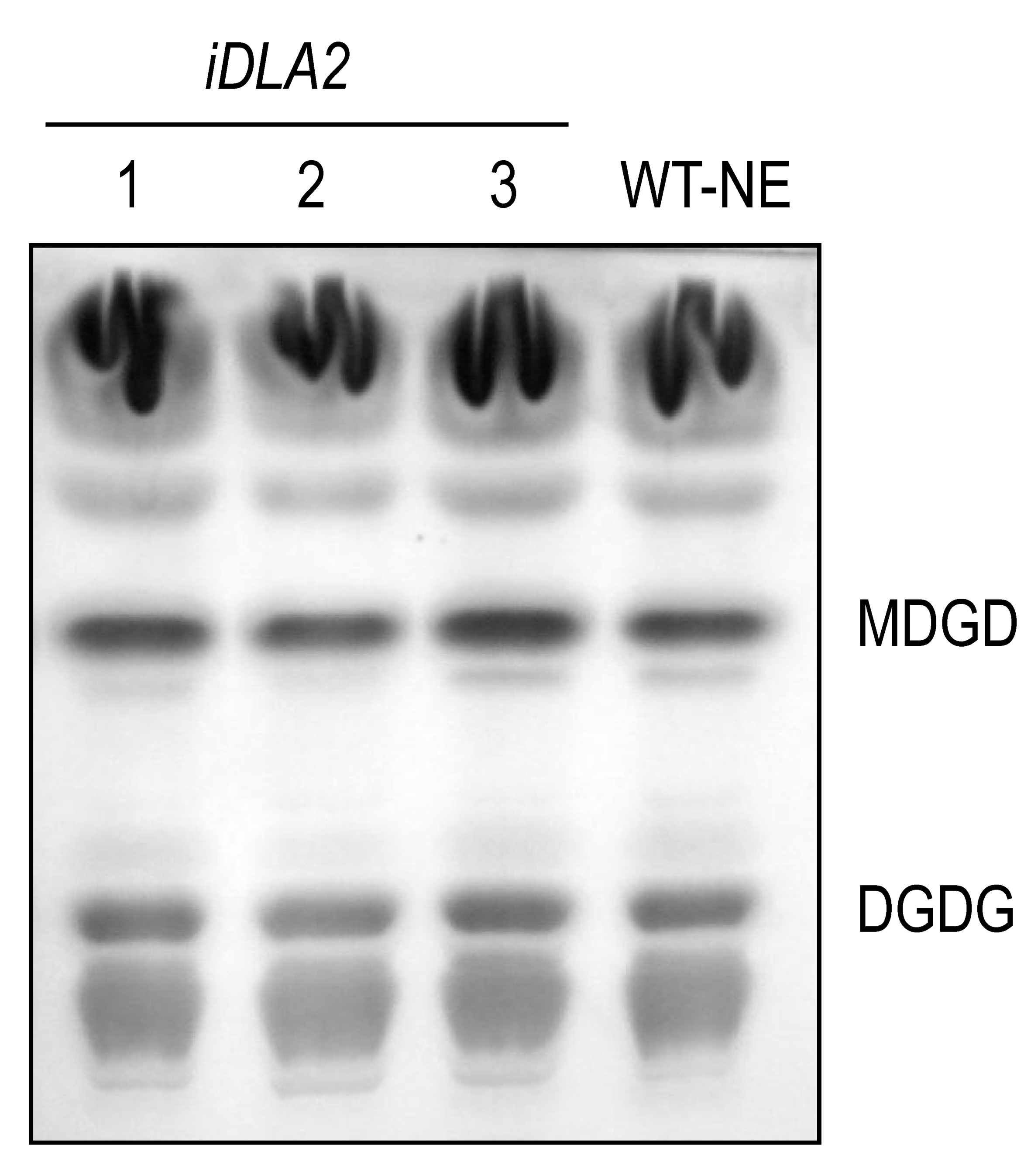

Supplement: Figure S6 — Lipid accumulation in iDLA2 lines. Lipids from whole cells were isolated according to Rengstl et al. [83] from 50 mL photoautotrophically grown wild-type transformed with the empty vector NE 537 (WT-NE) or DLA2-RNAi lines (iDLA2-1, -2, -3). After extraction, lipids were separated by thin-layer chromatography visualized by incineration. The mayor chloroplast lipids monogalactosyldiacylglycerol (MGDG) and digalactosyldiacylglycerol (DGDG) are indicated. (TIFF) [file pbio.1001482.s006.tiff]

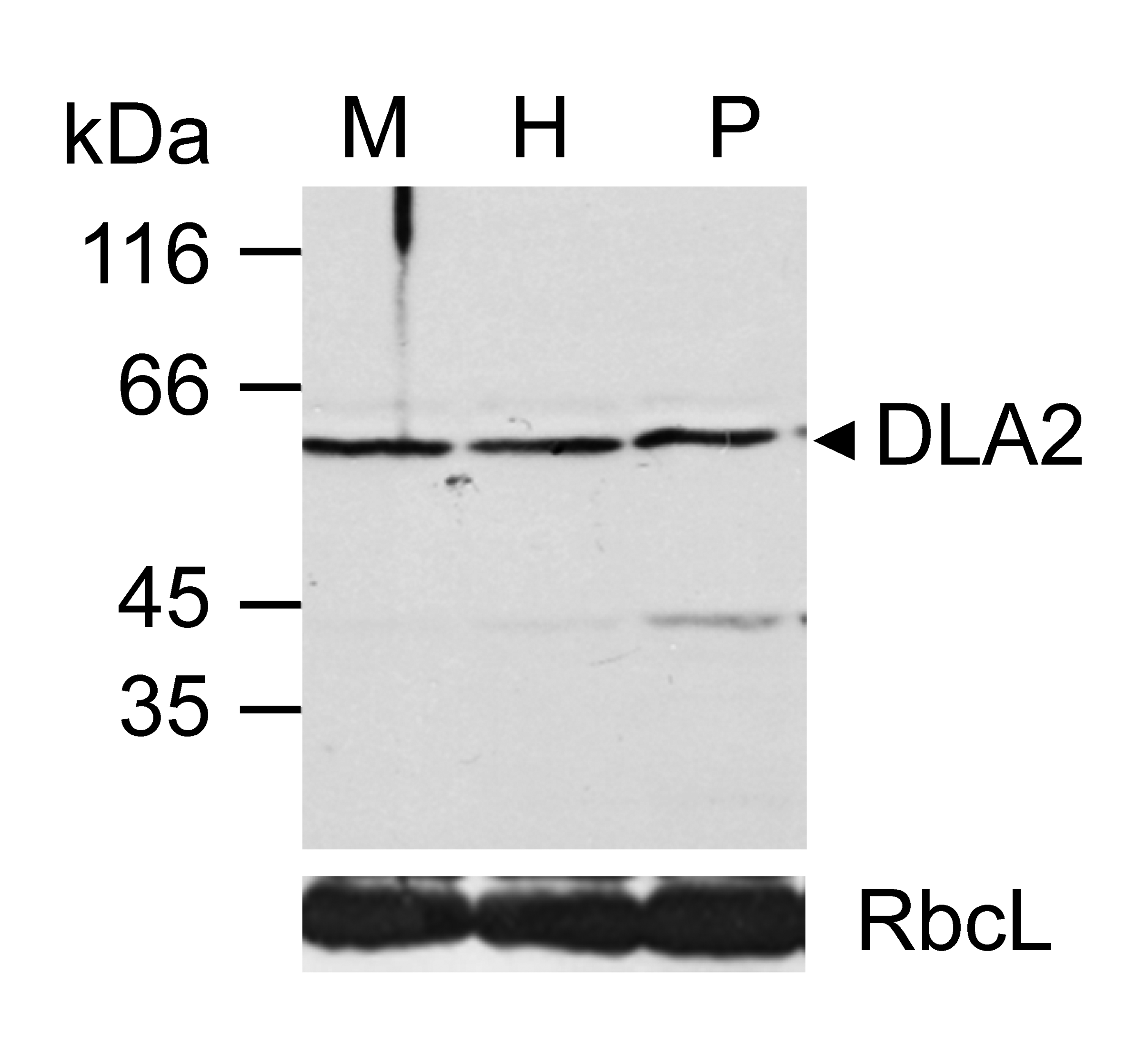

Supplement: Figure S7 — DLA2 protein accumulation under different growth conditions. A total of 30 µg of whole cell proteins from a cell-wall-deficient wild-type strain grown under mixotrophic (M), heterotrophic (H), or photoautotrophic (P) conditions were separated by SDS-PAGE and subjected to immunoblot analysis. The blot was probed with antibodies against DLA2 and the large subunit of Rubisco (RbcL). (TIF) [file pbio.1001482.s007.tif]

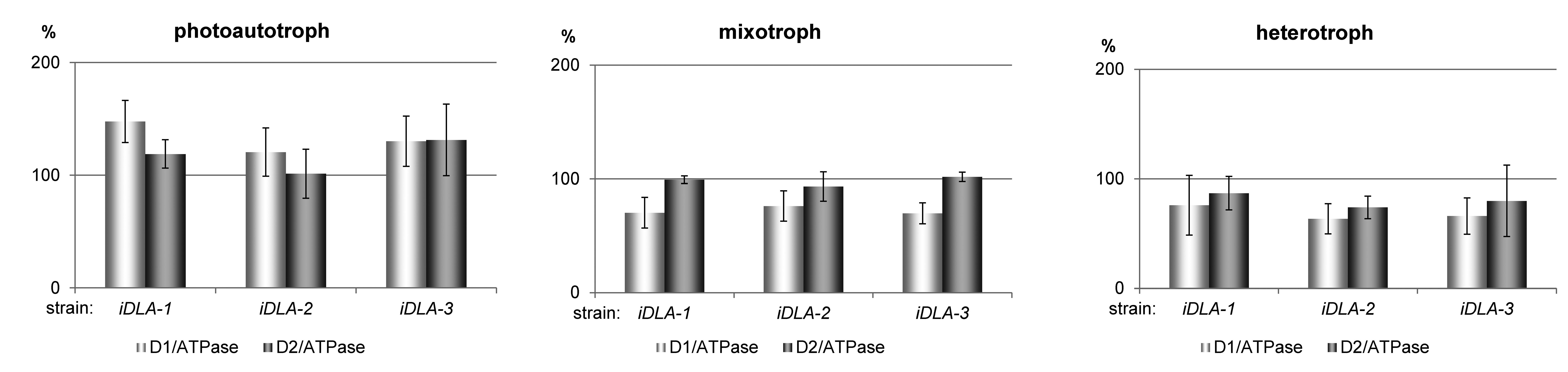

Supplement: Figure S8 — Quantification of D1 and D2 protein synthesis rates in DLA2–RNAi lines under various growth conditions. D1 and D2 protein synthesis rates were determined as described in Figure 7. Quantification of signals was performed by using AlphaEaseFC software (Alpha Innotech Corp.) and calculating the ratio of D1 or D2, respectively, over AtpA/B. Values obtained for WT-NE were set to 100%. Growth conditions as indicated. (TIFF) [file pbio.1001482.s008.tiff]

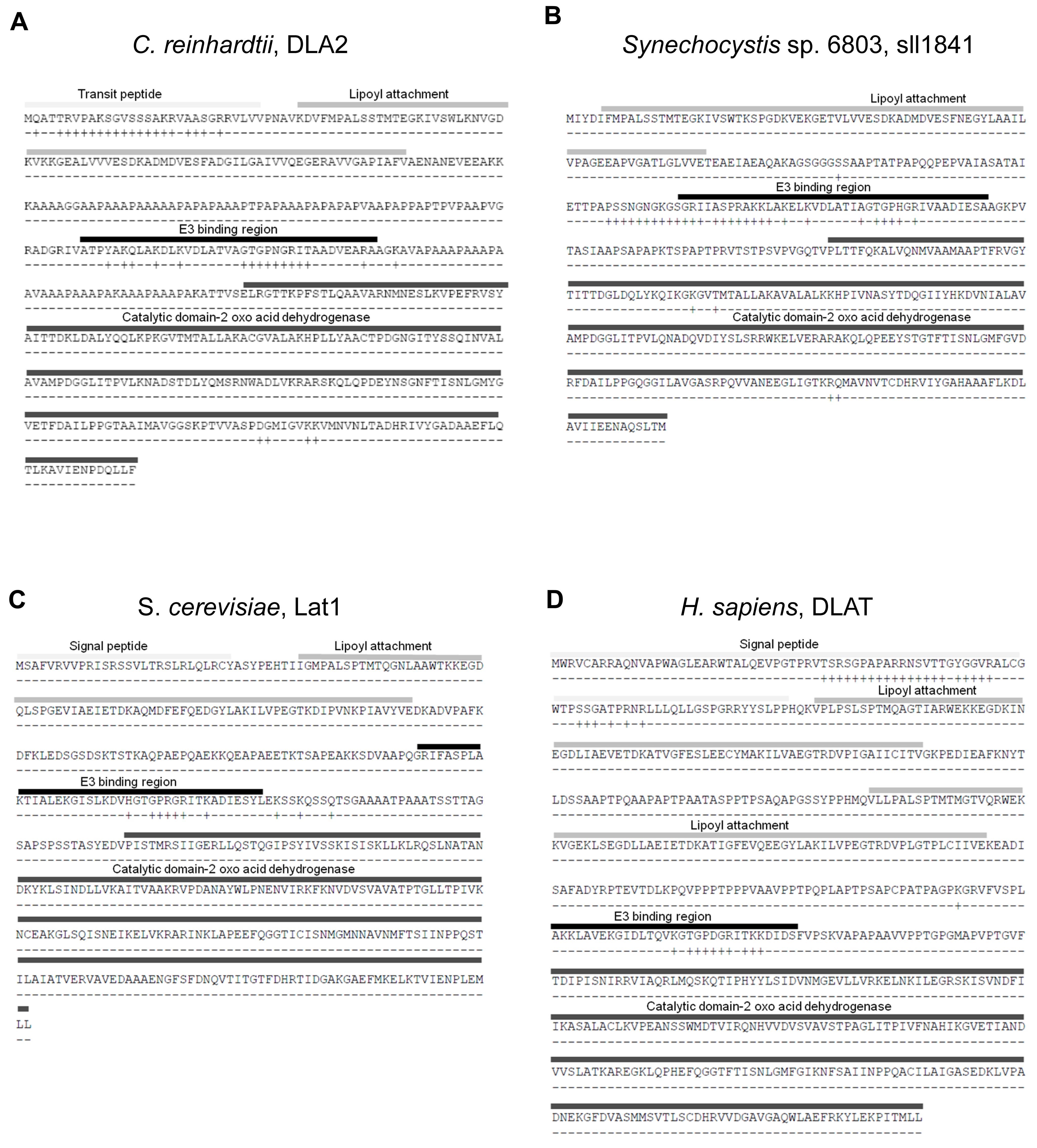

Supplement: Figure S9 — Prediction of RNA binding residues in E2 subunits of various organisms. Possible RNA binding residues within the amino acid sequence of E2 subunits from C. reinhardtii (A), Synechocystis sp. PCC 6803 (B), S. cerevisiae (C), and H. sapiens (D) were predicted by using the RNAbindR software with set “optimal prediction” (http://bindr.gdcb.iastate.edu/RNABindR) [84]. Plus signs below the amino acid sequences indicate the predicted RNA-binding residues, whereas minus signs indicate a low probability of RNA binding. The conserved domains for lipoyl attachment, E3 binding, and the 2-oxo acid dehydrogenase catalytic domain are marked above the sequence by grey, black, and dark grey bars, respectively. Transit and signal peptides as predicted by TargetP [23]. Accession numbers as in Figure 2. (TIFF) [file pbio.1001482.s009.tiff]

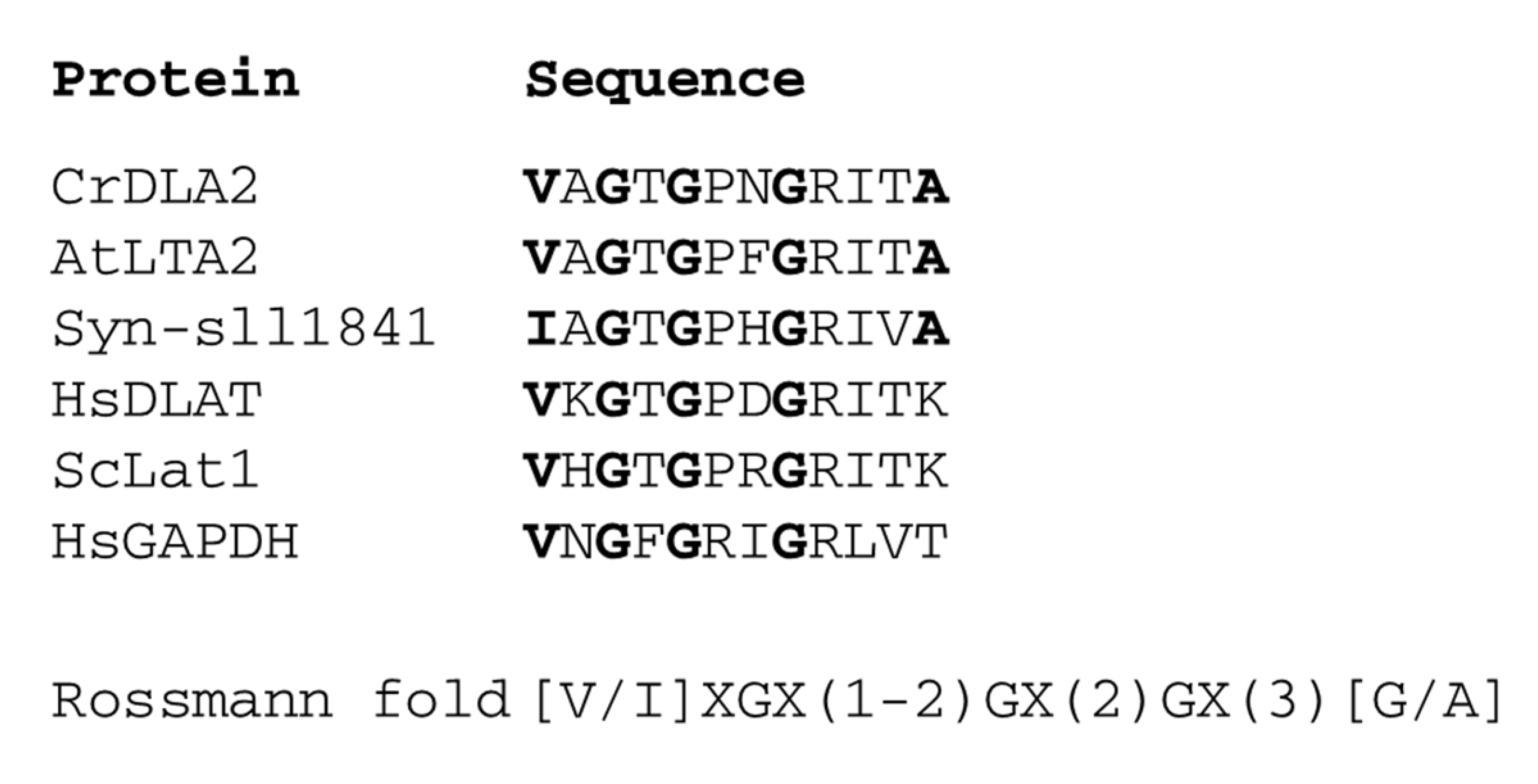

Supplement: Figure S10 — Alignment of predicted Rossmann folds within the amino acid sequence of PDC-E2 subunits and human GAPDH. For accession numbers, see Figure 2. H. sapiens, HsGAPDH (accession no: NP_002037). Highly conserved residues are written in bold. The Rossmann fold consensus sequence is shown below the alignment as given on the NIH MBI laboratory prediction server (http://nihserver.mbi.ucla.edu//cgi-bin/Rossmann/Rossmann.cgi). (TIFF) [file pbio.1001482.s010.tiff]
